# Integrated structural variation and point mutation signatures in cancer genomes using correlated topic models

**DOI:** 10.1371/journal.pcbi.1006799

**Published:** 2019-02-22

**Authors:** Tyler Funnell, Allen W. Zhang, Diljot Grewal, Steven McKinney, Ali Bashashati, Yi Kan Wang, Sohrab P. Shah

**Affiliations:** 1 Department of Epidemiology & Biostatistics, Memorial Sloan Kettering Cancer Center, New York, New York, United States of America; 2 Department of Molecular Oncology, BC Cancer Agency, Vancouver, British Columbia, Canada; 3 Department of Pathology and Laboratory Medicine, University of British Columbia, Vancouver, British Columbia, Canada; Helsingin Yliopisto, FINLAND

## Abstract

Mutation signatures in cancer genomes reflect endogenous and exogenous mutational processes, offering insights into tumour etiology, features for prognostic and biologic stratification and vulnerabilities to be exploited therapeutically. We present a novel machine learning formalism for improved signature inference, based on multi-modal correlated topic models (MMCTM) which can at once infer signatures from both single nucleotide and structural variation counts derived from cancer genome sequencing data. We exemplify the utility of our approach on two hormone driven, DNA repair deficient cancers: breast and ovary (n = 755 samples total). We show how introducing correlated structure both within and between modes of mutation can increase accuracy of signature discovery, particularly in the context of sparse data. Our study emphasizes the importance of integrating multiple mutation modes for signature discovery and patient stratification, and provides a statistical modeling framework to incorporate additional features of interest for future studies.

## Author summary

Over time DNA accumulates mutations from a variety of sources. Some mutations result from external mutagens, such as UV radiation, while others result from processes occurring within the cell itself. Each of these sources can impart characteristic patterns of mutations on the genome, known as mutation signatures, which can be detected using computational techniques. Loss of DNA repair mechanisms can leave specific mutation signatures in the genomes of cancer cells. To identify cancers with broken DNA-repair processes, accurate methods are needed for detecting mutation signatures and, in particular, their activities or probabilities within individual cancers. In this paper, we introduce a class of statistical modeling methods used for natural language processing, known as “topic models”, that outperform standard methods for signature analysis. We show that topic models that incorporate signature probability correlations across cancers perform best, while jointly analyzing multiple mutation types improves robustness to low mutation counts.

## Introduction

Patterns of mutation in cancer genomes reflect both endogenous and exogenous mutagenic processes [1], allowing inference of causative mechanisms, prognostic associations [2], and clinically actionable [3–6] vulnerabilities in tumors. Many mutational processes leave distinct genomic “footprints”, measurable via nucleotide substitution patterns [1], localised mutation densities, and patterns of structural variation (SV). As such, each mutagenic source (whether exogenous or endogenous) changes DNA in a characteristic manner, at genomic locations with preferred chemical and structural characteristics. Exogenous insults such as ultra-violet radiation and tobacco smoke-associated mutagens (*e.g*. benzo[a]pyrene) induce single nucleotide variants (SNVs) with characteristic C→T (at CC or TC dinucleotides) [7] and C→A mutation patterns [8], respectively; endogenous APOBEC activity mediates enzymatic 5-methylcytosine deamination, resulting in C→T substitution patterns at TC dinucleotides [7].

Cancer cells can also acquire endogenous mutator phenotypes, accumulating mutations [7] due to DNA repair deficiencies. Defective DNA repair processes induce both point mutations and structural variations [9], and include several mechanistic classes such as mismatch repair deficiency (MMRD), homologous recombination deficiency (HRD), microhomology mediated end-joining, and breakage fusion bridge processes. Defective DNA repair has been exploited in therapeutic regimes, including immune checkpoint blockade for mismatch repair deficiency [6], and synthetic lethal approaches for HRD [4, 5], underscoring their clinical importance.

Both point mutation signatures [10] and structural variation signatures [11] have been studied extensively as independent features of cancer genomes, mostly through non-negative matrix factorization (NMF) approaches [1, 3, 12–15]. As increasing numbers of whole genomes are generated from tumors in international consortia and focused investigator research, the need for robust signature inference methods is acute. Additional computational methods have been proposed [16–19], however no approaches jointly infer signatures from *both* point mutation and structural variations. We contend that systematic, integrative analysis of point mutation and structural variation processes enhances ability to exploit signatures for subgroup discovery, prognostic and therapeutic stratification, clinical prediction, and driver gene association.

Latent Dirichlet allocation (LDA) [20], a popular and effective approach for natural language document analysis, is well suited to the task of mutation signature inference. Although LDA was designed to extract topics from documents, these concepts can be mapped to mutation signatures and somatic mutation catalogues derived from tissue samples, respectively. In this paper we introduce the correlated topic model (CTM) [21], an extension of LDA which incorporates signature correlation, and a multi-modal correlated topic model (mf-CTM.dt in Salomatin *et al*. [22], hereafter referred to as MMCTM). A modality is a particular kind of data, and in this report SNV and SV counts are two distinct modalities. The MMCTM thereby jointly infers signatures from multiple mutation types, such as SNVs and SVs.

Signature correlations can arise through a mutational process generating multiple signatures, as with the HRD-associated SNV and SV signatures. C→T substitutions caused by APOBEC cytidine deaminases have also been shown to cluster around SV breakpoints [12]. Correlations between mechanistically independent signatures can also occur; for example, COSMIC SNV signatures 1 and 5 are both correlated with age of diagnosis in some cancer types [23].

We set out to investigate whether statistical modeling that could encode correlations between signatures could enhance accuracy in signature analysis. We show how integrating SNV and SV signature probability correlation improves mutation signature inference relative to NMF and standard topic modeling methods. By incorporating statistical correlation and multiple modalities, more information is provided to the model, improving inference further, while still maintaining distinct signatures for each modality.

Motivated by the need to better understand mutation signatures in the context of DNA repair deficiency, we analysed breast and ovarian tumour genomes. We applied the MMCTM to SNV and SV somatic mutations derived from whole genomes (breast [13] and ovarian [2]; 755 samples total), performing joint statistical inference of signatures. Our results reveal correlated topic models as an important analytic advance over standard approaches. Rigorous benchmarking over mutation signatures inferred from previously published mutation corpora was used to establish metrics for comparison. We show systematically how correlation integration improves inference, especially in the context of sparse mutation counts, and where SNVs and SVs are considered jointly. In addition, we report novel strata using MMCTM-derived signatures, including patient groups exhibiting combined whole genome SNV and SV signature profiles from breast and ovary cancers. We automatically recovered *BRCA1*-like and *BRCA2*-like homologous recombination repair deficient breast and ovarian cancers, where the tumors bearing the well known SNV HRD signature were reproducibly split on the basis of SVs. In aggregate, our study reveals the importance of simultaneously considering multiple classes of genomic disruption as a route to expanding mutation signature discovery, and their downstream impact on novel stratification across human cancers.

## Results

### Correlated topic models for signature inference

**Fig 1:**
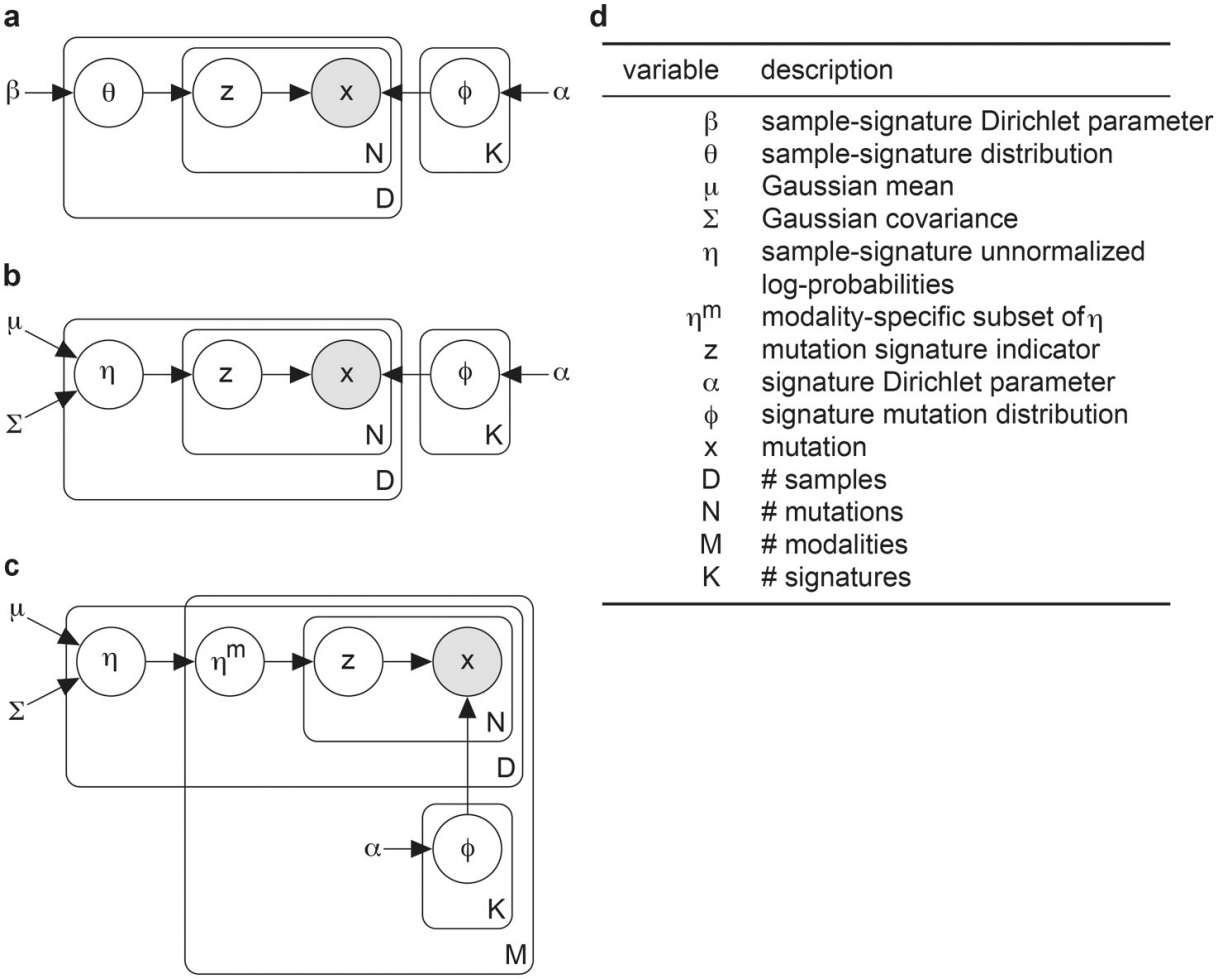
Plate notation for LDA, CTM, and MMCTM. Graphical models for the **a** LDA, **b** CTM and **c** MMCTM models, with **d** descriptions of their variables. See S1 Text for detailed descriptions.

We developed a suite of probabilistic correlated topic models (Fig 1) to evaluate their utility in signature discovery. We describe the models here briefly and refer to S1 Text for more detailed descriptions.

Topic models represent mutation signatures as discrete distributions over unique mutation categories (*e.g*. C→T substitutions at TCT trinucleotides). Each sample is then represented as a discrete distribution over signatures. How the sample-signature distributions are generated differ between LDA (Fig 1a and 1d) and the correlated topic models. In LDA, this variable is drawn from a Dirichlet distribution [20]. With the correlated topic models, however, it comes from the transformation of a variable that is distributed according to a multivariate Gaussian distribution [21] (Fig 1b, 1c and 1d). By using the multivariate Gaussian, the covariance of signature probabilities across samples can be captured. The multi-modal extension of the CTM (*i.e* MMCTM) encodes mutation counts and signatures for different modalities (*e.g*. SNVs and SVs) independently, except for the sample-signature probabilities which are all modeled using the same Gaussian distribution, allowing for cross-modality correlations.

We also developed a set of “independent” feature models based on the method introduced by Shiraishi *et al*. [16]—independent-feature LDA, CTM, and MMCTM (ILDA, ICTM, IMMCTM, S1 Fig, S1 Table, S1 Text). These models can treat each mutation feature (*e.g*. substitution type, flanking nucleotide) independently. That is, one feature for the mutation itself (say, C→T), and features for each piece of contextual information (*e.g*. 5′ A and 3′ G). Using this scheme, we drastically reduced the number of feature values: assuming 6 SNV types, and 2 flanking nucleotides the number of feature values is reduced from 6 * 4 * 4 = 96 to 6 + 4 + 4 = 14 [16].

### Datasets and feature construction

We studied mutation signatures in 560 breast [13] and 195 ovarian [2, 24] cancer genomes (S2 Table). Each dataset was analyzed separately to avoid biases from differences in sample sequencing, data-processing or annotation.

We constructed SNV features using the 6 types of pyrimidine-centric substitutions (C→A, C→G, C→T, T→A, T→C, T→G), and their flanking nucleotides. For example, a C→T substitution with an upstream A and downstream G is represented as the item “A[C→T]G”. We defined SV features by rearrangement type (deletion, tandem duplication, inversion, foldback inversion (FBI), translocation), number of homologous nucleotides around the breakpoints (0–1, 2–5, >5), and breakpoint distance (<10kbp, 10–100kbp, 100kbp–1Mbp, 1–10Mbp, >10Mbp, except for translocations). Foldback inversions are inverted duplications caused by breakage-fusion-bridge cycles.

We then computed counts of mutations, categorized as described above. The resulting count matrices were provided as input to LDA, CTM, MMCTM, and NMF (S1 Table).

### Correlated topic models improve signature inference

We compared NMF to the LDA, CTM, and MMCTM topic models. As NMF is commonly applied to normalized mutation counts, we also compared output from this alternative NMF procedure (NMF-NORM). Each method was run on input mutation counts constructed in an identical manner (*e.g*. for SNVS, 96 counts for each sample), and methods were compared using three different benchmarks: i) average per-mutation predictive log-likelihood; ii) logistic regression prediction accuracy of HRD labels; and iii) the mean absolute error of inferred solutions compared to a synthetic reference dataset.

For log-likelihood comparisons, we performed 5-fold cross validation, repeated 10 times, on the 560 breast cancer dataset. In each comparison, we fit SNV and SV signatures to four folds, leaving out a test fold (112 samples). We split mutation counts from each test fold sample into two parts, inferred sample-signature probabilities with one portion, and computed average per-mutation predictive log-likelihood values with the other portion. By evaluating each method on data different than those used for parameter estimation, we alleviated the risk of over-fitting parameters. This evaluation procedure only required estimated mutation signatures and sample-signature probabilities from each method, and did not depend on other model details, *e.g*. signature correlation structure. The average per-mutation predictive log-likelihood is an established comparison metric used in the topic modeling literature [25–27], and is also not directly optimized by any method here (unlike *e.g*. reconstruction error which is directly minimized by NMF). Although a likelihood-based metric may seem more applicable to the probabilistic models, NMF can be interpreted as maximum likelihood estimation of the “signature” and “activity” matrices under certain conditions (*e.g*. using Euclidean distance for the cost function maps NMF to a Gaussian emission model) [17, 28].

**Fig 2:**
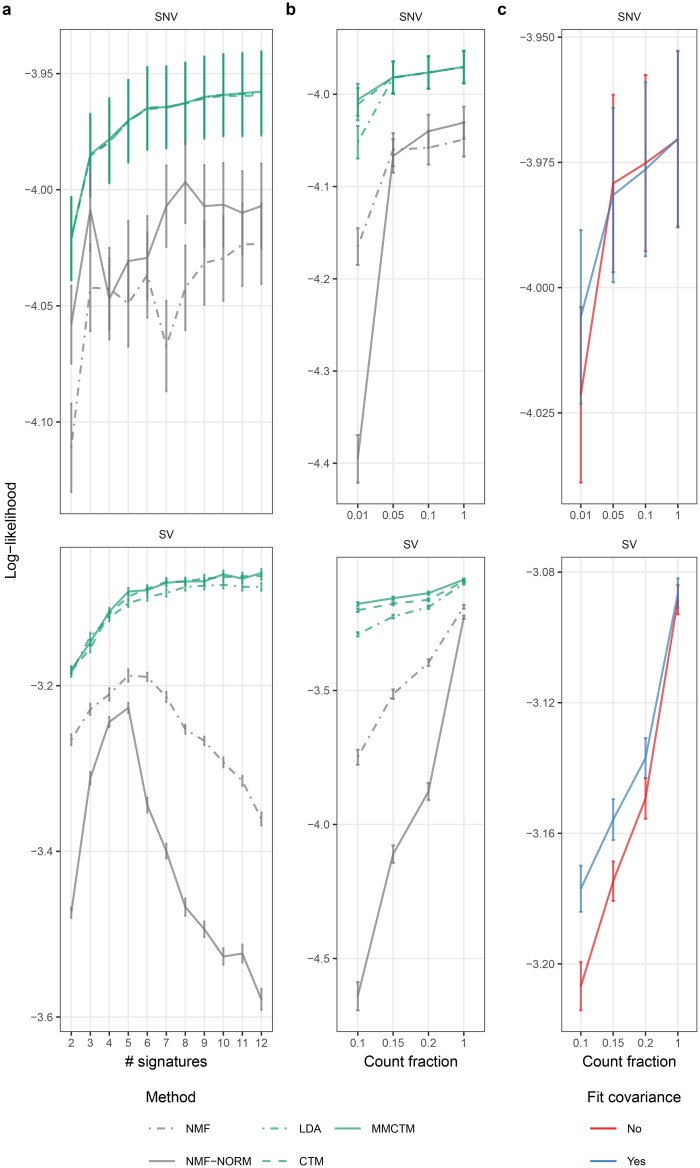
Predictive log-likelihood benchmark. SNV and SV signature per-mutation predictive log likelihood means ± standard error (n = 50) for: **a** 2–12 signatures, **b** a range of mutation count fractions, and **c** MMCTM with estimated or fixed Gaussian covariance matrix. NMF: applied to raw counts, NMF-norm: applied to normalized counts.

We first compared performance as a function of the number of signatures, fitting models over a range of 2–12 SNV and SV signatures (Fig 2a, S1 Dataset). For SNV signatures, LDA, CTM, and MMCTM performed similarly, and were consistently higher than the NMF methods across the full range of signature numbers. For SV signatures, the probabilistic topic models’ performance was consistently higher than the NMF models, and improved until a plateau was reached with an inflection point at 5. Within the topic models, the CTM and MMCTM showed better performance than LDA. NMF-NORM performance degraded with >5 signatures, and NMF performance degraded with >6 signatures.

Correlated topic models performed better than their non-correlated analogues at inferring SV signatures, possibly due to relatively low input counts for SV features. To explore this further, we compared performance over a range of mutation count fractions (Fig 2b, S2 Dataset). When subsetting SNV counts, LDA, CTM, and MMCTM performed roughly equally until only 1% of mutation counts were retained, at which point LDA performance became worse than the CTM and MMCTM. With fewer SV counts, the MMCTM performed better than the CTM, and both outperformed LDA. Importantly, correlated topic models were the least affected by reducing mutation counts, whereas NMF-NORM exhibited the worst performance decline, indicating that correlated models were in general more robust to data sparsity. Further, fixing the MMCTM covariance matrix during inference reduced it’s performance with fewer counts (Fig 2c, S2 Dataset), underlining the benefit of modeling signature correlations.

**Fig 3:**
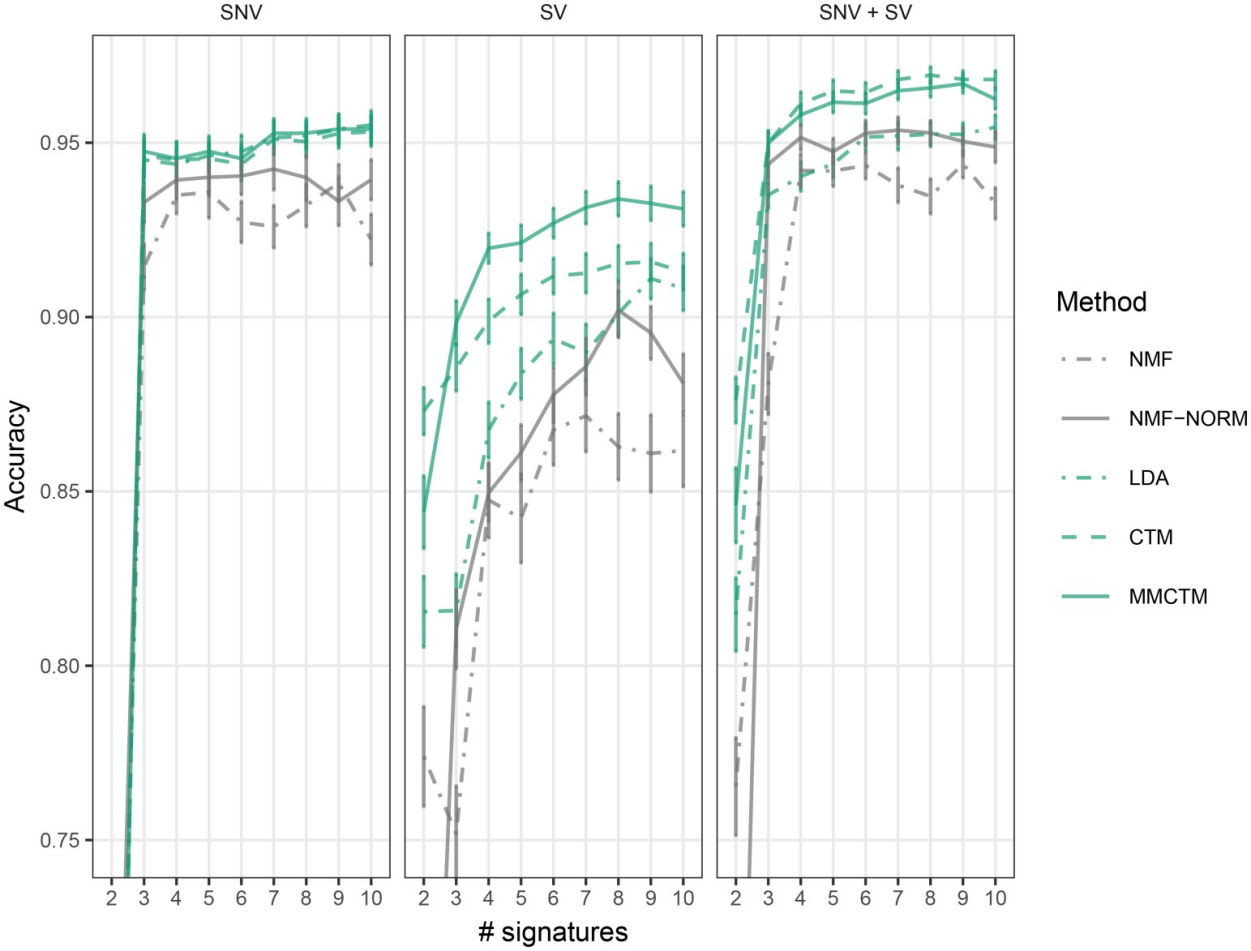
Low-dimensional classifier input benchmark. Accuracy means ± standard error (n = 50) is displayed for training with SNV (left), SV (middle), and both SNV and SV (right) signature probabilities. NMF: applied to raw counts, NMF-norm: applied to normalized counts.

We next compared the ability of these methods to provide informative, low-dimensional representations of samples, using signatures to stratify patients (Fig 3, S3 Dataset). We trained each method 10 times with random initializations on the full breast cancer dataset. We then trained a logistic regression classifier with the per-sample signature probabilities from each run as input features, and published labels from HRDetect [3]. HRD prediction accuracy scores were computed using 5-fold cross-validation. When the classifier was trained on only SNV signature probabilities, LDA, CTM, and MMCTM performed equally well. NMF and NMF-NORM generally performed worse. With SVs, the MMCTM signature probabilities provided the best accuracy, followed by the CTM and LDA. When the classifier was trained on both SNV and SV signature probabilities, the CTM and MMCTM performed better than other methods, further supporting the advantage of correlated models.

**Fig 4:**
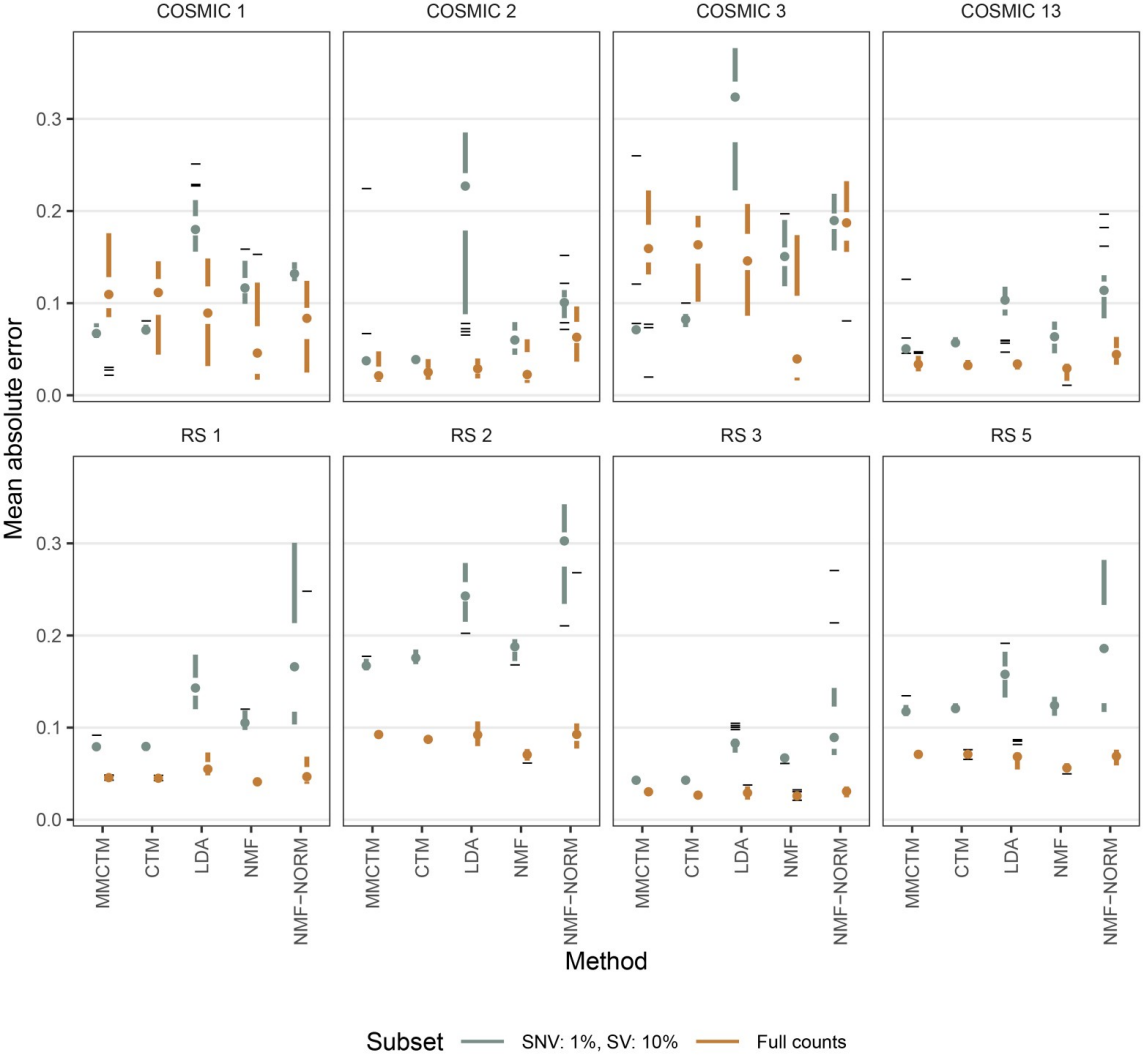
Signature probability mean absolute errors on synthetic data. Shown are mean absolute errors per method and per signature (n = 20) for estimated signature probabilities compared to reference probabilities. The experiment was repeated with full mutation counts and with 1% SNVs & 10% SVs. Data is represented as Tufte-like boxplots with the following elements: points (medians), gaps (first to third quartiles), whiskers (extend to the most extreme value no further than 1.5X the inter-quartile range from the gap edge), dashes (outliers). NMF: applied to raw counts, NMF-norm: applied to normalized counts.

We then tested each method on a simulated dataset based on SNV and SV counts from 560 breast cancers [13] (Fig 4, S2 Fig, S4 Dataset). Briefly, we used NMF to fit signature probabilities to a set of distinct SNV and SV signatures previously identified in this dataset (COSMIC 1, 2, 3, 13; RS 1, 2, 3, 5; see Methods) [13]. We note that using NMF-based signatures and estimated signature probabilities likely biased results in favour of NMF. Using the signatures, estimated signature probabilities, and mutation counts per sample, we generated 20 new sets of counts (560 synthetic tumour samples each) by sampling from a Poisson distribution. We then repeated the experiment by generating synthetic datasets with only 1% and 10% of the original SNVs and SVs. Signatures and signature probabilities were estimated using each method, selecting the best solution from 500 restarts, and the mean absolute error (MAE) was calculated between estimated and reference values.

While all methods generally performed well at recovering SNV signatures (all median MAE <0.01, except for LDA in COSMIC 2 with 1% counts), NMF-NORM performed worst at inferring SV signatures (adjusted t-test p-values <0.05, S2 Fig). The relatively low MAE even with reduced mutation counts also indicated that these methods are able to detect similar signatures as with a full set of mutations. Considering signature probabilities with full counts (Fig 4), NMF performed best for COSMIC 1 (except *v.s*. NMF-NORM), COSMIC 3, COSMIC 13, and the SV signatures, except *v.s*. CTM in RS 3 (adjusted t-test p-values <0.05). NMF-NORM was worst for COSMIC 2, 3, and 13 (adjusted t-test p-values <0.05). However, with 1% of the original SNV counts, the MMCTM did better than other methods for COSMIC 1, 3, & 13, and both the MMCTM and CTM did best for COSMIC 2 (adjusted t-test p-values <0.05). With 10% SV counts, the MMCTM did best for RS 2 and 5. The CTM and MMCTM both did better than other methods for RS 1 and 3 (adjusted t-test p-values <0.05).

The performances of the independent-feature models (ILDA, ICTM, IMMCTM) were also robust to low mutation counts, as previously described [16], and they typically worked well for SV signature estimation. However, they are generally worse than the MMCTM at inferring SNV signatures, and were not considered for subsequent analysis (S3 and S4 Figs).

Overall, correlated topic models produced superior predictive mutation signature distributions and low-dimensional representations of samples. This was especially true when each sample had few mutations, as for SVs. We also found similar patterns in log-likelihood comparisons using the smaller ovarian cancer dataset (S4 Fig), except we detected no major differences between the CTM and MMCTM. Performance of probabilistic topic models was stable across a range of topic hyperparameter values (S3d Fig), and across random restarts compared to NMF (S5 Fig), although randomization schemes differ across these two classes of methods.

### Integrated SNV and SV signatures in breast cancer

**Fig 5:**
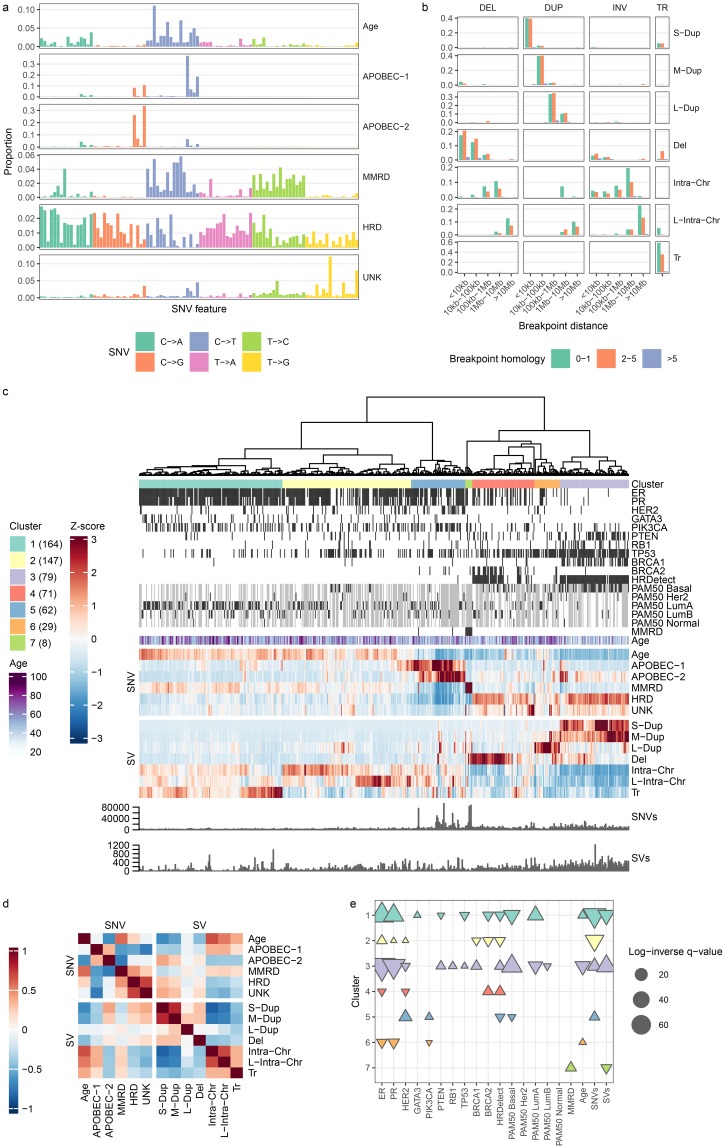
BRCA-EU mutation signature analysis. **a** SNV mutation signatures. SNVs are organized according to the SNV type (color). Within each type, SNVs are further organized into the pattern of flanking nucleotides (A—A, A—C, …,T—G, T—T). **b** SV mutation signatures. SVs are grouped by type (DEL: deletion, DUP: tandem duplication, INV: inversion, TR: translocation). **c** Heatmap of relative signature probabilities in BRCA-EU samples. Each heatmap column represents a single sample, and is composed of the SNV and SV signature probabilities output from the MMCTM model. The values for each signature (row) have been standardized, producing z-scores. Heatmap display has been truncated to ±3. Samples have been hierarchically clustered according to their transformed signature probabilities and cluster labels are indicated with colors underneath the dendrogram. The number of samples in each cluster is indicated in parentheses in the cluster legend. ER, PR, and HER2 positive status, BRCA1/2 mutation or methylation status, other gene driver mutation status, HRDetect prediction, and MMRD status is indicated with black bars. Grey cells represent missing data for annotation tracks. Samples with zero mutations for a mutation type also have grey signature probability cells. **d** Correlation heatmap between SNV and SV signatures. **e** Annotation associations for sample clusters. Upward- and downward-pointing triangles indicate enrichment and depletion, respectively. Adjusted p-values >0.05 are not shown. Colors correspond to cluster colors indicated in the heatmap.

We next analysed mutations from the 560 breast cancer genomes [13] with the MMCTM for stratification analysis (S6a Fig). We simultaneously fit 6 SNV and 7 SV signatures to counts of SNVs and SVs (Fig 5a and 5b, S7 Fig, S5 Dataset, see Methods for signature count selection). We found SNV signatures similar to those previously identified with proposed etiologies (S8 Fig), including the age-related (Age, COSMIC 1), APOBEC (APOBEC-1 & APOBEC-2, COSMIC 2 & 13), MMRD (COSMIC 20), and HRD (COSMIC 3) signatures. Additionally we found an SNV signature of unknown etiology, UNK (COSMIC 17). We identified SV signatures including small, medium, and large tandem duplications (S-Dup, M-Dup, L-Dup), deletions (Del), intrachromosomal SVs (Intra-Chr & L-Intra-Chr), and translocations (Tr).

Some signatures were more likely to co-occur in the same tumour, possibly reflecting common etiology. According to the MMCTM model, the two APOBEC signatures were positively correlated (Pearson’s r = 0.34) (Fig 5d, S6 Dataset), and the HRD SNV signature was positively correlated with the S-Dup signature (r = 0.3), as expected. The Age signature was positively correlated with Intra-Chr (r = 0.66), L-Intra-Chr (r = 0.53), and Tr (r = 0.38) SV signatures.

We next performed unsupervised clustering over tumours on joint per-tumour SNV and SV signature probabilities (Fig 5c, S6b and S9 Figs, S7 and S8 Dataset, see Methods). The resulting 7 groups included two (clusters 1 & 2, n = 164 & 147) enriched for the Age signature (see S10a Fig, S9 Dataset for significant cluster-signature associations). Cluster 1 was enriched for the Tr signature, and both clusters 1 & 2 were enriched for Intra-Chr and L-Intra-Chr. While the Age signature was most correlated with patient age at diagnosis (r = 0.23, adjusted p-value << 0.0001), Intra-Chr was second most correlated (r = 0.20, adjusted p-value << 0.0001). Cluster 1 was associated with Luminal A cancers with relatively fewer SNVs, and contained tumours from generally older patients (see Fig 5e, S10 Dataset for significant cluster-annotation associations). This implies that older patients may be more likely to have accumulated SVs in their cancers’ etiology as function of background rates, indicating a putative SV-related age signature for breast cancer.

We also observed clusters with *BRCA1*/*BRCA2* mutations and methylation (clusters 3 & 4, n = 79 & 71), as previously described [13]. These tumours typically exhibited an HRD phenotype, and had elevated probability of the HRD SNV signature. Cluster 3 was associated with the S-Dup & M-Dup SV signatures, and more *BRCA1*, *RB1*, and *PTEN* driver mutations than expected by chance. As expected, cluster 3 patients were predominantly from the Basal PAM50 class. Cluster 4 was associated with the Del signature, and *BRCA2* mutation. In contrast to cluster 1, patients in cluster 3 also tended to be younger than patients in other clusters. The majority (87%) of *BRCA1/2* samples fell into clusters 3 & 4, although *BRCA1/2* mutant tumours that fell outside these clusters often had evidence of HRD, albeit with increased probability of unrelated signatures (*e.g*. L-Dup in cluster 6). Of patients predicted by HRDetect [3] to harbour HRD, 97% fell within the *BRCA1/2* (clusters 3 & 4) groups, demonstrating that the MMCTM output provides a substrate upon which known biological clusters are recovered, with further stratification as a result of SNV and SV integration.

Cluster 5 (n = 62) was enriched for the APOBEC-1, APOBEC-2, Intra-Chr, and L-Intra-Chr signatures, and was also enriched for *HER2*-positive tumours, relating Her2-amplification and APOBEC deamination processes for approximately 11% of breast cancers, as previously reported [29]. Cluster 6 (n = 29) was the only group enriched for L-Dup, and also contained older patients than expected by chance. Cluster 7 (n = 8) was associated with defective DNA mismatch repair (MMRD), and the MMRD SNV signature, consistent with previous reports [30].

### SNV and SV signature probabilities segregate ovarian cancer samples into prognostically distinct groups

A recent analysis of ovarian tumours revealed a novel high-grade serous ovarian carcinoma (HGSC) sub-group with relatively worse prognosis, characterized by increased frequency of foldback inversions (FBI) [2]. Their analysis combined NMF-based SNV signature analysis with ad-hoc SV and copy number variant (CNV) features. Here we expanded on some of their findings using the MMCTM on a merged data set consisting of 133 samples from Wang et al. [2] and 62 samples from the International Cancer Genome Consortium (ICGC) ovarian cancer whole genome dataset [31].

**Fig 6:**
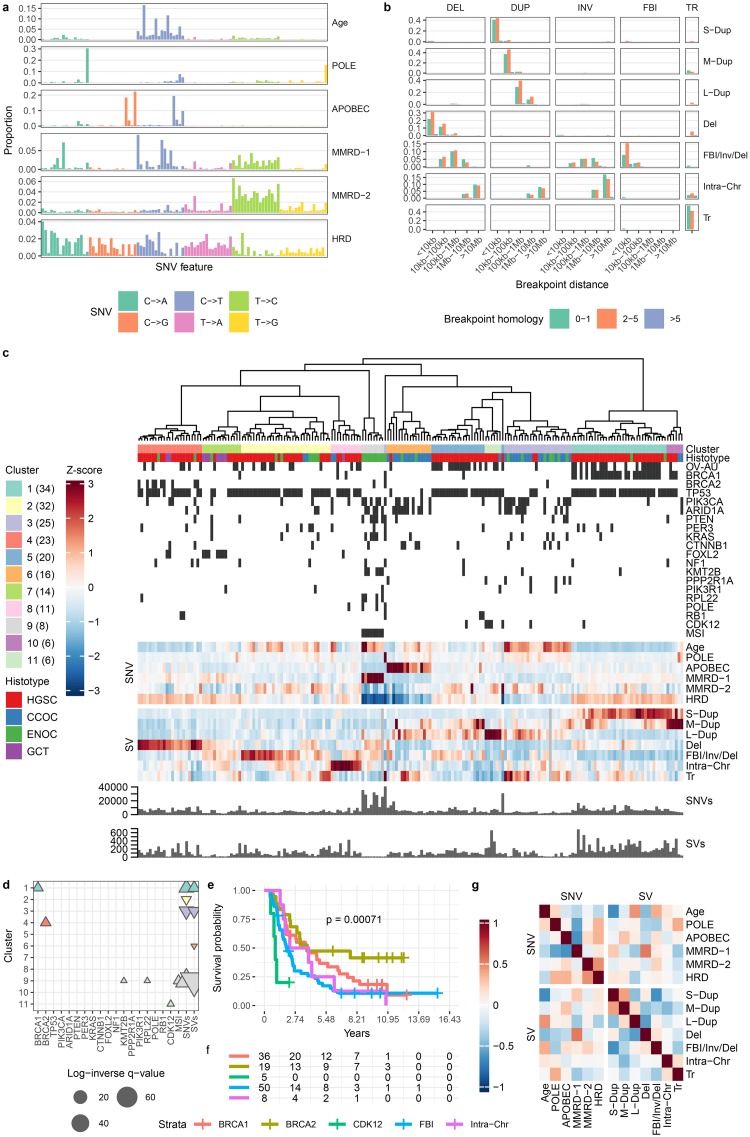
Ovarian cancer mutation signature analysis. **a** SNV mutation signatures. SNVs are organized according to the SNV type (color). Within each type, SNVs are further organized into the pattern of flanking nucleotides (A—A, A—C, …,T—G, T—T). **b** SV mutation signatures. SVs are grouped by type (DEL: deletion, DUP: tandem duplication, INV: inversion, FBI: foldback inversion, TR: translocation). **c** Heatmap of relative signature probabilities in ovarian cancer samples. Each heatmap column represents a single sample, and is composed of the SNV and SV signature probabilities output from the MMCTM model. The values for each signature (row) have been standardized, producing z-scores. Heatmap display has been truncated to ±3. Samples have been hierarchically clustered according to their transformed signature probabilities and cluster labels are indicated with colors underneath the dendrogram. The number of samples in each cluster is indicated in parentheses in the cluster legend. Samples from the ICGC OV-AU project are indicated with black bars, as is microsatellite instability (MSI) and gene mutation status. Samples with zero mutations for a mutation type also have greyed signature probability cells. The number of SNVs for a *POLE* mutant sample has been truncated to 40k in the barplot; The actual number is 596,135. **d** Annotation associations for sample clusters. Upward- and downward-pointing triangles indicate enrichment and depletion, respectively. Adjusted p-values >0.05 are not shown. Colors correspond to cluster colors indicated in the heatmap. **e** Kaplan-Meier curves for HGSC samples only. **f** Risk table for HGSC samples only. Kaplan-Meier curve plots and risk tables share x-axes. **g** Correlation heatmap between SNV and SV signatures.

We fit 6 SNV and 7 SV signatures to mutation counts from the 195 ovarian cancer genomes (Fig 6a and 6b, S11 Fig, S5 Dataset, see Methods for signature count selection), including endometrioid carcinomas (ENOC), clear cell carcinomas (CCOC), granulosa cell tumours (GCT), and HGSC (S2 Table). Amongst the resultant SNV signatures were the previously described Age (COSMIC 1), APOBEC (COSMIC 13), HRD (COSMIC 3), MMRD-1 (COSMIC 20), MMRD-2 (COSMIC 26), and *POLE* (COSMIC 10) signatures (S8 Fig, see also for a comparison to the breast SNV signatures). The SVs included signatures for small, medium, and large tandem duplications (S-Dup, M-Dup, L-Dup); deletions (Del); FBI, inversions, and deletions (FBI/Inv/Del); intrachromosomal SVs (Intra-Chr); and translocations (Tr). The association of deletions with FBI can be understood in terms of the underlying cause of FBI: breakage-fusion-bridge cycles. After the loss of a telomere, sister chromatids fuse and are then pulled apart during mitosis, producing one chromosome with a foldback inversion and another with a terminal deletion.

We clustered the tumours according to their joint standardized SNV and SV signature probabilities, which resulted in 11 groups (Fig 6c, S12 Fig, S7 and S8 Dataset). While the original study identified one HRD signature group [2], our analysis here produced two major HRD clusters (1 & 4, n = 34 & 23), roughly defined by tumours with S-Dup and M-Dup (see S10 Fig, S9 Dataset for cluster-signature associations) coupled with loss of *BRCA1* (see Fig 6d, S10 Dataset for cluster-annotation associations), and small deletions (Del) coupled with loss of *BRCA2*, respectively. The association of *BRCA1/2* status with tandem duplication and deletion SV signatures has been reported in breast cancer tumours [13], and was reflected in our analysis of the 560 breast cancer dataset (Fig 5, described above), providing strong evidence for *BRCA1*-like and *BRCA2*-like HRD sub-strata crossing tumour types.

Cluster 2 (n = 32), 5 (n = 20), 7 (n = 14), and 9 (n = 8) were all enriched for the FBI/Inv/Del signature. Cluster 9 also included all microsatellite instable (MSI) ENOC tumours, and was also associated with MMRD-1, Age, and Del signatures, along with higher numbers of SNVs, and *KMT2B* and *RPL22* mutations. Cluster 3 (n = 25) contained mainly CCOC and ENOC tumours enriched for the Age, L-Dup, and Tr signatures. Cluster 6 (n = 16) included tumours highly enriched for APOBEC signature probability. Cluster 7 (n = 14) was associated with the HRD SNV signature as well as Del and FBI/Inv/Del. Cluster 8 (n = 11) was only enriched for the Intra-Chr signature. Cluster 10 (n = 6) was similar to the *BRCA1* cluster (1), but was more strongly associated with the M-Dup signature. Another small cluster of mainly HGSC tumours (11, n = 6), was associated with higher probability of the L-Dup signature, and *CDK12* mutations, an association supported by a previous study [32].

By inspecting the signature correlations output by the MMCTM model (Fig 6g, S6 Dataset) we saw that the HRD SNV signature was positively correlated with the S-Dup (r = 0.12) signature, as may be expected from the underlying biology of these signatures. The Age signature is positively correlated with the L-Dup (r = 0.45) and FBI/Inv/Del (r = 0.37) signatures. MMRD-1 is positively correlated with the S-Dup (r = 0.26), and Del (r = 0.53) SV signatures.

HGSC patient groups, defined by their standardized mutation signature probabilities, differed in survival rates. We defined 5 HGSC groups (see Methods), representing *BRCA1*-mutant (clusters 1, 10; n = 36), *BRCA2*-mutant (cluster 4, n = 19), FBI (clusters 2, 5, 7; n = 50), Intra-Chr (cluster 8, n = 8), and *CDK12*-like tandem duplicator tumours (cluster 11, n = 5). We compared overall-survival amongst the HGSC super-clusters using the Kaplan-Meier method (Fig 6e and 6f). The *BRCA2*/deletion cluster had the highest survival rate, while the *CDK12*/tandem duplicator group had the worst. Comparing the HGSC clusters in a pairwise fashion, the *CDK12* group had worse survival than the *BRCA1* and *BRCA2* groups (adjusted log-rank p-value < 0.05). The FBI group had worse survival than the *BRCA2* group (adjusted log-rank p-value < 0.05). The *BRCA1*/tandem-duplication group had an intermediate survival rate, but the survival curve was not significantly different than those of the FBI or *BRCA2* groups (adjusted log-rank p > 0.05). While FBI was previously identified as a marker for poor prognosis [2], activity of a mutational process linked with loss of *CDK12* and producing 100kbp–1Mbp tandem duplications could indicate even worse outcomes. Overall, the MMCTM analysis represented a refinement of signature-based prognostic stratification in HGSC indicating *BRCA2*-like HRD as the best performing group of patients, followed by *BRCA1*-like HRD, Intra-Chr, FBI, and *CDK12*-like tandem duplicators.

### MMCTM produces reproducible mutation signatures

To evaluate the reproducibility of signatures inferred using the MMCTM, we applied the method to the two independent HGSC datasets included in our ovarian cancer analysis above. Specifically, 59 samples previously published by our group, and 62 samples from ICGC. Each HGSC group contained signatures that were similar between both groups, including HRD associated SNV and SV signatures. Both groups also showed a segregation of *BRCA1*- and *BRCA2*-like cases based on per-sample signature probabilities (S13 and S14 Figs).

We also compared SNV signatures inferred in the ovarian and breast datasets to each other and to the COSMIC signatures (S8 Fig). In both datasets we found signatures similar to those previously reported to occur in ovary and breast cancers [2, 10, 13], including the APOBEC; HRD; and age-associated signatures, demonstrating the ability of the MMCTM to capture established signatures.

## Discussion

Through integrated statistical inference and analysis of SNV and SV mutation signatures, our results reveal at once correlated signatures and patient stratification within DNA repair deficient tumours. Our findings have several implications for the field. The use of structural variations in signature analysis is less common than for point mutations, in part due to the relative paucity of whole-genome sequencing datasets. Here, we show the significant new value from their joint interpretation, and set the framework for their simultaneous consideration across a broad range of tumour types. Moreover, our results demonstrate that correlated statistical modeling improves signature inference in the context of sparse mutation counts. The HRD point mutational signature is well described, but automated association of tandem duplications within *BRCA1*-like and interstitial deletions within *BRCA2*-like cancers represents an important refinement, reproduced here in two independent cancer types, with data from two independent studies. Furthermore, we show in the ovarian cancer cohort how this has prognostic implication, superseding what could be derived from gene-based biomarkers (i.e. if only *BRCA1* and *BRCA2* mutation status were considered).

We have introduced a new formalism for mutation signature analysis in cancer genomes. Our approach models the correlation between signatures, which provides their performance increase. However, when no correlations exist between signature probabilities, this method will likely not provide much benefit. In these situations, a researcher may opt to use an alternative, such as NMF or LDA. Nevertheless, signature correlations exist in at least breast and ovarian cancer, as shown in this report, and we believe analysis of other cancer types will benefit from our approach.

The topic models discussed in this manuscript produce signature probabilities, as opposed to activity estimates, which requires a subtle difference in interpretation. Signature probabilities are related to activities, but they indicate the probability of signatures generating a mutation, rather than the proportion of mutations generated by a signature. The topic models discussed output non-zero signature probabilities for each sample, due to their Bayesian formulation. Since every sample is unlikely to have experienced activity from every detected signature, one may wish to set a probability threshold to determine active signatures for downstream analysis. However, the optimal choice of probability threshold is a matter for future investigation.

Correlated topic models are significantly more robust to reduced mutation burden, which can occur in a number of scenarios. We have already described that signature extraction from SVs, at the level detected in the breast and ovarian datasets analysed here, benefits from correlated signature modeling. Analysis of other low-count mutation types may also benefit, for example mutations called from exome or single-cell sequencing experiments.

Importantly, the statistical framework of the MMCTM is flexible and extensible. While here we show the advantage of integrated SNV and SV analysis, the MMCTM can seamlessly integrate other count-based features such as copy number events, double strand breaks, and telomeric insertions. As the field develops, we suggest a robust and extensible framework will be required to encode and integrate multiple feature types of the genome as they relate to mutational processes.

The advantage of our relatively simple SNV and SV integration is evident and motivates further advances through multi-modal statistical modelling leading to richer biological interpretations of endogenous and potentially exogenous processes. In conclusion, our findings reinforce the importance of an integrated, holistic view of multiple classes of genomic scarring to drive discovery and characterization of mutation processes across human cancers.

## Materials and methods

### Mutation data processing

Nucleotides flanking SNVs were extracted from human reference GRCh37. The number of each type of SNV (*e.g*. C→T) with a particular flanking sequence was counted. SV calls were split according to type (deletion, tandem duplication, inversion, foldback-inversion, translocation), the level of homology (0–1, 2–5, >5 bp), and breakpoint distance (<10kbp, 10–100kbp, 100 kbp–1Mbp, 1–10Mbp, >10Mbp), then counted. Foldback inversion calls were not included in the breast cancer dataset. Breakpoint distance bins are those used in a previous study on SV signatures [13]. Breakpoint distance was not calculated for translocations, as the concept is not applicable for this class of SVs. SNV and SV counts per sample were computed from the mutations used for signature analysis. Additional ovary sample gene mutation annotations were computed from SNV and indel calls according to the original paper.

### Inference in topic models and NMF

For LDA and ILDA, parameters were inferred using mean-field Variational Bayes. For CTM, MMCTM, ICTM and IMMCTM, parameter inference was performed using mean-field variational EM. The MMCTM updates and derivations can be found in Salomatin et al. [22]. See S1 Text for detailed descriptions of the topic models.

When using only a single mutation type, the MMCTM reduces to the CTM described by Blei and Lafferty [21] (similarly for the IMMCTM and ICTM). Therefore, the CTM and ICTM parameters were inferred using the MMCTM and IMMCTM implementations, but with counts from a single mutation type. The CTM, ICTM, LDA, ILDA, and NMF methods were used to compute SNV or SV signatures separately.

The probabilistic topic models were implemented similarly using the Julia language v0.6.3 [33]. NMF models were fit using the coordinate descent solver implementation in the Scikit-learn library [34] v0.19.1.

### Method comparison

NMF was run on both raw and normalized mutation counts. Normalization was performed by dividing mutation counts by sample totals, for each mutation type.

For log-likelihood-based comparisons, mutation counts were split according to a stratified 10 × 5 cross validation scheme; For each histotype, samples were split into 5 training and test sets. The splitting procedure was performed 10 times, resulting in 50 training and test sets.

Each method was run on each training set and evaluated on each corresponding test set, using random initialization. Random initialization for the topic models involved generating random positive integer values for the variational signature-mutation dirichlet parameters. Evaluation was performed by randomly splitting the mutations in each test sample into observed and hidden sets. Signature probabilities for each test sample were estimated using the observed test mutation counts, then the per-mutation predictive log likelihood was computed using the hidden test mutation counts. Methods were tested over a range of 2–12 signatures, as well as over a range of count subsets. Multi-modal topic models were given the same number of signatures for SNVs and SVs. An additional, similar comparison was performed by fitting the MMCTM to this data with covariance fixed to the identity matrix.

Count subset comparisons were performed by removing mutations from each genome, retaining only a given fraction. Mutations were randomly selected according to their type (*e.g*. C(C→T)T) and relative type proportions. These mutations were removed and the genome mutation counts updated. The updated mutation counts were then input to the compared methods. SNVs were subset to 1, 5, 10%, while keeping SVs at 100%. SVs were subset to 10, 15, 20%, while keeping SNVs at 100%. For the breast cancer dataset, the number of SNV and SV signatures was fixed at 5, selected by observing the log-likelihood curves in the above benchmarking experiment (S3 Fig) with the objective of choosing a “fair” value. For the ovarian cancer dataset, the number of SNV and SV signatures was fixed to 6 and 5, respectively.

The stability of method solutions were also compared over 100 random restarts on 4/5 of the breast cancer dataset. Solutions were evaluated on the remaining 1/5 of the samples in the manner described earlier.

Predictive log likelihoods were computed on test sets with signatures for SNVs and SVs separately. The likelihood computation involves the signatures fit with the training data, sample-signature probabilities estimated using the observed test counts, and the hidden test counts. The average per-mutation predictive log likelihood for a particular mutation type is given in Eq 1.

$$l=\frac{\sum_{d}^{D}\sum_{n}^{N_{d}}\text{log}(\sum_{k}^{K}p\left(X_{n}^{d}|\phi_{k}\right)p\left(Z_{n}^{d}=k|\theta_{d}\right))}{\sum_{d}^{D}N_{d}}$$ (1)

where *D* is the number of samples, *N*_*d*_ is the number of mutations in sample *d*, *K* is the number of signatures, *X* is the mutations in sample *d*, *Z* is the mutation-signature indicators, *ϕ*_*k*_ is the signature-mutation distribution, and *θ*_*d*_ is the sample-signature distribution.

For comparisons involving the breast cancer dataset, foldback inversion counts were not provided to NMF as these SV types were not included in this dataset. When evaluating the NMF solutions, the outputs are normalized to produce valid probability distributions that can be used for the log-likelihood calculations. Since NMF does not take into account uncertainty during estimation, the sum of probabilities calculation above can occasionally produce zeros. To avoid taking log(0), we add 10^−16^ to the sum of probabilities for NMF. Topic model signature-mutation and sample-signature distribution point-estimates were obtained by taking the mean of their variational posterior distributions.

For the logistic regression classifier-based comparisons, each signature detection method was trained 10 times with 2–10 signatures, using the full 560 breast cancer dataset. For multi-modal methods, the same number of SNV and SV signatures was given. The sample-signature distributions were used as training data for the classifier along with previously published HRDetect-derived labels. HRDetect negative cases were subsampled for each method run to produce balanced datasets for training and evaluation, with 124 positive and negative labels each. Three types of tests were performed: using only SNV, only SV, or both SNV and SV sample-signature distributions. Stratified 5-fold cross-validation was performed for each test, resulting in 5 × 10 = 50 scores for each method, training data type, and setting of the number of signatures. The output score of cross validation is the mean accuracy of the logistic regression classifier. Parameter inference was performed using the Scikit-learn [34] v0.19.1 implementation with the liblinear solver and maximum 10,000 iterations.

Simulated datasets were generated by first selecting COSMIC SNV signatures 1, 2, 3, 13, and breast cancer SV signatures [13] RS 1, 2, 3, and 5. These SNV signatures were reported as present in the breast cancer dataset [13], and they are qualitatively distinct from each other. SV signatures largely defined by clustered breakpoints were excluded as that feature was not included in this analysis. Reference signature probabilities were estimated using NMF, the given signatures, and counts for the 560 breast cancer dataset. 10 synthetic datasets were generated, where for each mutation type in each sample, counts were generated by drawing from a Poisson distribution with rate equal to the number of mutations in the sample multiplied by the reference signature matrix and the sample’s signature probability vector. This approach is similar to that used in a previous study [18]. This procedure was repeated using the reference signatures, signature probabilities, and mutation counts subsetted to 1% SNVs and 10% SVs. Signatures and signature probabilities per dataset were then estimated by running each method 500 times with random restarts and choosing the best solution per method based on predictive log-likelihood. Topic model signature hyperparameters were set to 1.0. Estimated signatures were then matched to the reference signatures, and the mean absolute differences between the reference and estimated values were computed. Signature matching was performed by finding the pairwise combination of estimated and reference signatures that gave the lowest mean absolute error. Then the matching procedure was repeated for the rest of the signatures, while ignoring previously assigned reference signatures.

### Choosing the number of signatures

The number of signatures to estimate in the breast and ovarian datasets was selected by inspecting the log-likelihood curves from the benchmarking experiment, using the elbow curve method (S15 Fig). The number of signatures to estimate in the two HGSC datasets was selected by fitting the MMCTM to approximately half the mutations in each sample, and computing the average per-mutation log-likelihood on the other half of the mutations. This differs from the benchmarking cross-validation scheme in that it takes in account all samples in the dataset.

### Fitting MMCTM to cancer datasets for downstream analysis

The model was initially fit to each dataset 1000 times for a limited number of iterations. *α* hyper-parameters were set to 0.1. Each restart is run until the relative difference in predictive log likelihood on the training data was < 10^−4^ between iterations. The restart with the best mean rank of the SNV and SV predictive log likelihoods was selected for fitting to convergence with a tolerance of 10^−5^.

### Sample hierarchical clustering

Samples were clustered using sample-signature probabilities for SNV and SV signatures together. Signature probabilities were converted to Z-scores for each signature across samples. By standardizing the probabilities, the inter-sample differences of low-prevalence signatures are given increased emphasis relative to higher-prevalence signatures. Hierarchical agglomerative clustering was performed using the Euclidean metric, and Ward linkage. Discrete clusters were formed using the R dynamicTreeCut package [35] v1.63 with method = “hybrid”, deepSplit = FALSE, and minClusterSize = 3.

### Sample cluster enrichment and depletion tests

Enrichment of a sample cluster’s signature probability was tested using an unequal variance one-sided t-test against the signature probabilities of other clusters.

For the breast cancer dataset, cluster associations with ER, PR, HER2, MMRD, and PAM50 status were performed with a two-tailed Fisher’s exact test. Differences in Age or the number of SNVs and SVs were tested with two-tailed unequal variance t-tests. Driver gene mutation and HRDetect prediction associations were computed using a blocked permutation test.

The permutation tests were performed as follows: For each cluster, “new” clusters were generated by sampling tumour samples without replacement from the full dataset. New clusters maintained the same ER, PR, and HER2 status composition as the original cluster. The difference in proportions of samples with the annotation of interest between the new cluster and all other samples was computed. Two-tailed p-values were calculated using Eq 2:

$$p=\frac{1+\sum_{n}^{N}I\left(abs\left(s^{\prime }\right)\geq abs\left(s\right)\right)}{1+N}$$ (2)

where *N* is the number of permutations (generated clusters, here 10,000), and *s* is the statistic of interest for the original cluster (*e.g*. difference in proportions of samples with loss of *TP53*), and *s*′ is the same statistic for a generated cluster. This procedure attempts to correct for correlations between the tested annotations and ER, PR, and HER2 status.

Gene mutation status and MSI cluster associations in ovarian cancer were tested with the blocked permutation test described above, accounting for histotype rather than ER, PR, and HER2 status. Differences in SNV and SV counts were performed with two-tailed unequal variance t-tests. Due to the presence of a *POLE* mutant sample with a very high number of SNVs, t-tests for this statistic were performed on count ranks. The unequal variance t-test on ranked data is a robust alternative to Student’s t-test and the Mann-Whitney U test when assumptions are violated [36].

Cluster-signature and cluster-annotation p-values within each dataset were corrected using the Benjamini & Hochberg method [37].

### Survival analysis

HGSC samples grouped according to the hierarchical clustering were compared by estimating overall-survival Kaplan-Meier curves for each cluster, using the R survival package. Clusters 2, 5, and 7 were grouped as they were all enriched for the FBI/Inv/Del signature, and had no significant difference in survival outcome. We call this the “FBI” group. Similarly, cluster 10 was grouped with cluster 1 as it contained *BRCA1* mutant patients with similar signature profiles. P-values were calculated using the log-rank test. Pairwise survival curve comparison p-values were adjusted using the Benjamini & Hochberg method [37] implemented in the R p.adjust function.

### Code availability

Topic model code is available in a GitHub repository: https://github.com/shahcompbio/MultiModalMuSig.jl.

## Supporting information

S1 Fig Plate notation for ILDA, ICTM, and IMMCTM. Graphical models for the **a** ILDA, **b** ICTM and **c** IMMCTM models, with **d** descriptions of their variables. See S1 Text for detailed descriptions.
(PDF)

S2 Fig Signature mean absolute errors on synthetic data. Shown are mean absolute errors per method and per signature for estimated signatures compared to the reference signatures. The experiment was repeated with full mutation counts and with 1% SNVs & 10% SVs. Data is represented as Tufte-like boxplots with the following elements: points (median), gap (first to third quartile), whisker (extends to the most extreme value no further than 1.5X the inter-quartile range from the gap edge), dash (outlier). NMF: applied to raw counts, NMF-norm: applied to normalized counts.
(PDF)

S3 Fig Comparisons of NMF, LDA, CTM, MMCTM, ILDA, ICTM, and IMMCTM, using the 560 breast cancer dataset. Displayed are SNV and SV signature log likelihood means ± standard error for: **a** 2–12 signatures, and **b** a range of mutation count fractions. **c** Logistic regression accuracy means ± standard error for predicting HRD labels using per-sample signature probabilities across a range of 1–10 signatures. **d** Method comparison across topic Dirichlet hyperparameter values using the breast cancer dataset. Displayed are log likelihood means ± standard error. NMF: applied to raw counts, NMF-norm: applied to normalized counts. Vertices and error bars are dodged slightly to reduce overplotting.
(PDF)

S4 Fig Comparison of NMF with LDA, CTM, MMCTM, ILDA, ICTM, and IMMCTM, using the ovarian cancer dataset. Displayed are log likelihood means ± standard error for: **a** 2–15 signatures, and **b** a range of mutation count fractions. Top panels are evaluations on SNV counts, bottom panels are evaluations on SV counts only. NMF: applied to raw counts, NMF-norm: applied to normalized counts. Downsampled SNV: only SNV counts are down-sampled. Downsampled SV: only SV counts are down-sampled. The down-sampling fractions are different for SNV and SV counts. Vertices and error bars are dodged slightly to reduce overplotting.
(PDF)

S5 Fig Log likelihoods across random restarts. Average per-mutation predictive log-likelihoods from 100 restarts for SNV and SV signatures inferred by each method. Values have been mean-centered.
(PDF)

S6 Fig Mutation processes and mutation signature analysis workflow. **a** Analysis workflow for the multimodal topic models MMCTM and IMMCTM. **b** Mutation process activity is detected as patterns of mutations, *i.e*. mutation signatures, in the genome. Samples with common levels of signature probabilities may be grouped, and potentially exhibit similar phenotypes.
(PDF)

S7 Fig SNV signatures for the 560 genomes BRCA-EU dataset. Mutation and flanking sequence shown on x-axis.
(PDF)

S8 Fig Binary heatmap indicating which SNV signatures have cosine similarity ≥ 0.8. Included are SNV signatures from COSMIC, the breast, and ovarian cancer datasets.
(PDF)

S9 Fig Heatmap of relative probabilities of signatures in BRCA-EU samples. Each heatmap column represents a single sample, and is composed of the probabilities of SNV and SV signatures output from the MMCTM model. The values for each signature (row) have been standardized, producing z-scores. Heatmap display has been truncated to ±3. Samples have been hierarchically clustered according to their transformed signature probabilities and cluster labels are indicated with colours underneath the dendrogram. The number of samples in each cluster is indicated in parentheses in the cluster legend. ER, PR, and HER2 positive status are indicated with black bars. Similarly, *BRCA1/2* mutation status and HRDetect prediction are indicated.
(PDF)

S10 Fig Sample cluster signature probability comparisons. Tests compared signature probability means for clusters in the **a** breast, and **b** ovarian cancer datasets. Adjusted p-values >0.05 are not shown. Cluster labels are colored according to those in the associated signature probability heatmap.
(PDF)

S11 Fig SNV signatures for the ovarian cancer dataset. Mutation and flanking sequence shown on x-axis.
(PDF)

S12 Fig Heatmap of relative probabilities of signatures in ovarian cancer samples. Each heatmap column represents a single sample, and is composed of the probabilities of SNV and SV signatures output from the MMCTM model. The values for each signature (row) have been standardized, producing z-scores. Heatmap display has been truncated to ±3. Samples have been hierarchically clustered according to their transformed signature probabilities and cluster labels are indicated with colours underneath the dendrogram. The number of samples in each cluster is indicated in parentheses in the cluster legend. Samples from the ICGC OV-AU project are indicated with black bars, as is gene mutation and MSI status.
(PDF)

S13 Fig Shah HGSC cancer mutation signature analysis. **a** SNV mutation signatures. SNVs are organized according to the SNV type (color). Within each type, SNVs are further organized into the pattern of flanking nucleotides (A—A, A—C, …,T—G, T—T). **b** SV mutation signatures. SVs are grouped by type (DEL: deletion, DUP: tandem duplication, INV: inversion, FBI: foldback inversion, TR: translocation). **c** Heatmap of relative signature probabilities in HGSC cancer samples. Each heatmap column represents a single sample, and is composed of the probabilities of SNV and SV signatures output from the MMCTM model. The values for each signature (row) have been standardized, producing z-scores. Heatmap display has been truncated to ±3. Samples have been hierarchically clustered according to their transformed signature probabilities and cluster labels are indicated with colors underneath the dendrogram. The number of samples in each cluster is indicated in parentheses in the cluster legend. Samples with mutated *BRCA1/2* or methylated *BRCA1* genes indicated with black boxes under the cluster assignments above the heatmap. **d** Signature log likelihood means ± standard error for 2–12 signatures. Signatures estimated from one half of counts, log-likelihood evaluated on the other half. Used to choose number of signatures.
(PDF)

S14 Fig ICGC HGSC cancer mutation signature analysis. **a** SNV mutation signatures. SNVs are organized according to the SNV type (color). Within each type, SNVs are further organized into the pattern of flanking nucleotides (A—A, A—C, …,T—G, T—T). **b** SV mutation signatures. SVs are grouped by type (DEL: deletion, DUP: tandem duplication, INV: inversion, FBI: foldback inversion, TR: translocation). **c** Heatmap of relative signature probabilities in HGSC cancer samples. Each heatmap column represents a single sample, and is composed of the probabilities of SNV and SV signatures output from the MMCTM model. The values for each signature (row) have been standardized, producing z-scores. Heatmap display has been truncated to ±3. Samples have been hierarchically clustered according to their transformed signature probabilities and cluster labels are indicated with colors underneath the dendrogram. The number of samples in each cluster is indicated in parentheses in the cluster legend. Samples with mutated or methylated *BRCA1/2* genes indicated with black boxes under the cluster assignments above the heatmap. **d** Signature log likelihood means ± standard error for 2–12 signatures. Signatures estimated from one half of counts, log-likelihood evaluated on the other half. Used to choose number of signatures.
(PDF)

S15 Fig MMCTM SNV and SV log likelihood means ± standard error across signature number. Shown for: **a** breast, and **b** ovarian cancer datasets. Signature number choice indicated as an green vertical line.
(PDF)

S1 Table Description of mutation signature methods. (PDF)

S2 Table Dataset breakdown. (PDF)

S1 Text Descriptions of the topic models. (PDF)

S1 Dataset Method benchmarking log-likelihood values across a range of the number of signatures. Columns: method (signature inference method; string), evaluation (snv or sv; string), k (number of signatures; integer), n (cross validation repeat; integer), fold (cross validation fold; integer), ll (log-likelihood; float), dataset (breast, ovary; string).
(TSV)

S2 Dataset Method benchmarking log-likelihood values across a range of mutation count fractions. Columns: method (signature inference method; string), evaluation (snv or sv; string), k (number of signatures; integer), snv_frac (fraction of retained SNVs; float), sv_frac (fraction of retained SVs; float), n (cross validation repeat; integer), fold (cross validation fold; integer), ll (log-likelihood; float), dataset (breast, ovary; string).
(TSV)

S3 Dataset Method benchmarking logistic regression accuracy across a range of the number of signatures. Columns: score (logistic regression accuracy; float), k (number of signatures; integer), n (cross validation repeat; integer), fold (cross validation fold; integer), method (signature inference method; string), train (training set, either SNV, SV or SNV & SV; string).
(TSV)

S4 Dataset Method benchmarking mean absolute error on synthetic breast cancer data. Columns: value (estimated value type, signature or probability; string), method (signature inference method; string), evaluation (snv or sv; string), signature (signature name; string), subset (1.0-1.0: full counts, 0.01-0.1: 1% SNVs & 10% SVs; string), seed (random seed; integer), mae (mean absolute error between ground truth and estimated value; float).
(TSV)

S5 Dataset Mutation signatures. Columns: modality (1: SNV, 2: SV; integer), signature (signature label; string), value (mutation term number; integer), term (mutation term; string), probability (signature-mutation probability; float), dataset (breast, ovary; string).
(TSV)

S6 Dataset Mutation signature probability correlations. Columns: signature_* (mutation signature label; string), correlation (sample-signature probability correlation between two signatures; float), dataset (breast, ovary; string).
(TSV)

S7 Dataset Mutation signature probabilities per sample. Columns: signature (mutation signature label; string), sample (sample id; string), probability (sample-signature probability; float), dataset (breast, ovary; string).
(TSV)

S8 Dataset Sample clusters. Columns: sample (sample id; string), cluster (cluster number; integer), dataset (breast, ovary; string).
(TSV)

S9 Dataset Sample cluster signature probability enrichment p-values. Columns: cluster (sample cluster; integer), signature (mutation signature label; string), p_value (enrichment p-value, float), mean_diff (difference between means, float), conf_low (lower bound of confidence interval; float), conf_high (upper bound of confidence interval; float), q_value (BH adjusted p-value; float), dataset (breast, ovary; string).
(TSV)

S10 Dataset Sample cluster annotation association p-values. Columns: label (annotation label; string), p_value (enrichment p-value, float), diff (difference between group statistics, float), conf_low (lower bound of confidence interval; float), conf_high (upper bound of confidence interval; float), test (statistical test; string), cluster (sample cluster; integer), q_value (BH adjusted p-value; float), dataset (breast, ovary; string).
(TSV)

## References

[1] Alexandrov LB, Nik-Zainal S, Wedge DC, Campbell PJ, Stratton MR. Deciphering signatures of mutational processes operative in human cancer. Cell reports. 2013;3(1):246–259. 10.1016/j.celrep.2012.12.008 23318258 PMC3588146

[2] Wang YK, Bashashati A, Anglesio MS, Cochrane DR, Grewal DS, Ha G, et al Genomic consequences of aberrant DNA repair mechanisms stratify ovarian cancer histotypes. Nature Genetics. 2017 10.1038/ng.3849 28436987

[3] Davies H, Glodzik D, Morganella S, Yates LR, Staaf J, Zou X, et al HRDetect is a predictor of BRCA1 and BRCA2 deficiency based on mutational signatures. Nature Medicine. 2017 10.1038/nm.4292 PMC5833945 28288110

[4] Swisher EM, Lin KK, Oza AM, Scott CL, Giordano H, Sun J, et al Rucaparib in relapsed, platinum-sensitive high-grade ovarian carcinoma (ARIEL2 Part 1): an international, multicentre, open-label, phase 2 trial. The Lancet Oncology. 2017;18(1):75–87. 10.1016/S1470-2045(16)30559-9 27908594

[5] Mirza MR, Monk BJ, Herrstedt J, Oza AM, Mahner S, Redondo A, et al Niraparib Maintenance Therapy in Platinum-Sensitive, Recurrent Ovarian Cancer. The New England journal of medicine. 2016;375(22):2154–2164. 10.1056/NEJMoa1611310 27717299

[6] Le DT, Uram JN, Wang H, Bartlett BR, Kemberling H, Eyring AD, et al PD-1 Blockade in Tumors with Mismatch-Repair Deficiency. The New England journal of medicine. 2015;372(26):2509–2520. 10.1056/NEJMoa1500596 26028255 PMC4481136

[7] Roberts SA, Gordenin DA. Hypermutation in human cancer genomes: footprints and mechanisms. Nature Reviews Cancer. 2014;14(12):786–800. 10.1038/nrc3816 25568919 PMC4280484

[8] Alexandrov LB, Ju YS, Haase K, Van Loo P, Martincorena I, Nik-Zainal S, et al Mutational signatures associated with tobacco smoking in human cancer. Science (New York, NY). 2016;354(6312):618–622. 10.1126/science.aag0299 PMC6141049 27811275

[9] Helleday T, Eshtad S, Nik-Zainal S. Mechanisms underlying mutational signatures in human cancers. Nature Reviews Genetics. 2014;15(9):585–598. 10.1038/nrg3729 24981601 PMC6044419

[10] Alexandrov LB, Nik-Zainal S, Wedge DC, Aparicio SA, Behjati S, Biankin AV, et al Signatures of mutational processes in human cancer. Nature. 2013;500(7463):415–421. 10.1038/nature12477 23945592 PMC3776390

[11] Yang L, Luquette LJ, Gehlenborg N, Xi R, Haseley PS, Hsieh CH, et al Diverse mechanisms of somatic structural variations in human cancer genomes. Cell. 2013;153(4):919–929. 10.1016/j.cell.2013.04.010 23663786 PMC3704973

[12] Nik-Zainal S, Alexandrov LB, Wedge DC, Van Loo P, Greenman CD, Raine K, et al Mutational processes molding the genomes of 21 breast cancers. Cell. 2012;149(5):979–993. 10.1016/j.cell.2012.04.024 22608084 PMC3414841

[13] Nik-Zainal S, Davies H, Staaf J, Ramakrishna M, Glodzik D, Zou X, et al Landscape of somatic mutations in 560 breast cancer whole-genome sequences. Nature. 2016;534(7605):47–54. 10.1038/nature17676 27135926 PMC4910866

[14] Gehring JS, Fischer B, Lawrence M, Huber W. SomaticSignatures: inferring mutational signatures from single-nucleotide variants. Bioinformatics. 2015;31(22):3673–3675. 10.1093/bioinformatics/btv408 26163694 PMC4817139

[15] Macintyre G, Goranova T, De Silva D, Ennis D, Piskorz AM, Eldridge M, et al Copy-number signatures and mutational processes in ovarian carcinoma. bioRxiv. 2017; p. 174201. 10.1038/s41588-018-0179-8 PMC6130818 30104763

[16] Shiraishi Y, Tremmel G, Miyano S, Stephens M. A simple model-based approach to inferring and visualizing cancer mutation signatures. PLoS genetics. 2015;11(12):e1005657 10.1371/journal.pgen.1005657 26630308 PMC4667891

[17] Fischer A, Illingworth CJ, Campbell PJ, Mustonen V. EMu: probabilistic inference of mutational processes and their localization in the cancer genome. Genome biology. 2013;14(4):R39 10.1186/gb-2013-14-4-r39 23628380 PMC3663107

[18] Rosales RA, Drummond RD, Valieris R, Dias-Neto E, da Silva IT. signeR: an empirical Bayesian approach to mutational signature discovery. Bioinformatics. 2016;33(1):8–16. 10.1093/bioinformatics/btw572 27591080

[19] Rosenthal R, McGranahan N, Herrero J, Taylor BS, Swanton C. DeconstructSigs: delineating mutational processes in single tumors distinguishes DNA repair deficiencies and patterns of carcinoma evolution. Genome biology. 2016;17(1):31 10.1186/s13059-016-0893-4 26899170 PMC4762164

[20] Blei DM, Ng AY, Jordan MI. Latent dirichlet allocation. Journal of machine Learning research. 2003;3(Jan):993–1022.

[21] Blei D, Lafferty J. Correlated topic models. Advances in neural information processing systems. 2006;18:147.

[22] Salomatin K, Yang Y, Lad A. Multi-field Correlated Topic Modeling In: SDM. SIAM; 2009 p. 628–637.

[23] Alexandrov LB, Jones PH, Wedge DC, Sale JE, Campbell PJ, Nik-Zainal S, et al Clock-like mutational processes in human somatic cells. Nature genetics. 2015;47(12):1402 10.1038/ng.3441 26551669 PMC4783858

[24] Consortium ICG, et al International network of cancer genome projects. Nature. 2010;464(7291):993 10.1038/nature08987 20393554 PMC2902243

[25] Wang C, Paisley J, Blei D. Online variational inference for the hierarchical Dirichlet process. In: Proceedings of the Fourteenth International Conference on Artificial Intelligence and Statistics; 2011. p. 752–760.

[26] Hoffman MD, Blei DM, Wang C, Paisley J. Stochastic variational inference. The Journal of Machine Learning Research. 2013;14(1):1303–1347.

[27] Wang C, Blei DM, et al A general method for robust Bayesian modeling. Bayesian Analysis. 2018.

[28] Févotte C, Cemgil AT. Nonnegative matrix factorizations as probabilistic inference in composite models. In: Signal Processing Conference, 2009 17th European. IEEE; 2009. p. 1913–1917.

[29] Roberts SA, Lawrence MS, Klimczak LJ, Grimm SA, Fargo D, Stojanov P, et al An APOBEC cytidine deaminase mutagenesis pattern is widespread in human cancers. Nature genetics. 2013;45(9):970–976. 10.1038/ng.2702 23852170 PMC3789062

[30] Davies H, Morganella S, Purdie CA, Jang SJ, Borgen E, Russnes H, et al Whole-Genome Sequencing Reveals Breast Cancers with Mismatch Repair Deficiency. Cancer research. 2017;77(18):4755–4762. 10.1158/0008-5472.CAN-17-1083 28904067

[31] Patch AM, Christie EL, Etemadmoghadam D, Garsed DW, George J, Fereday S, et al Whole—genome characterization of chemoresistant ovarian cancer. Nature. 10.1038/nature14410 26017449

[32] Popova T, Manié E, Boeva V, Battistella A, Goundiam O, Smith NK, et al Ovarian cancers harboring inactivating mutations in CDK12 display a distinct genomic instability pattern characterized by large tandem duplications. Cancer research. 2016;76(7):1882–1891. 10.1158/0008-5472.CAN-15-2128 26787835

[33] Bezanson J, Edelman A, Karpinski S, Shah VB. Julia: A fresh approach to numerical computing. SIAM Review. 2017;59(1):65–98. 10.1137/141000671

[34] Pedregosa F, Varoquaux G, Gramfort A, Michel V, Thirion B, Grisel O, et al Scikit-learn: Machine learning in Python. Journal of Machine Learning Research. 2011;12(Oct):2825–2830.

[35] Langfelder P, Zhang B, Horvath S. Defining clusters from a hierarchical cluster tree: the Dynamic Tree Cut package for R. Bioinformatics. 2007;24(5):719–720. 10.1093/bioinformatics/btm563 18024473

[36] Ruxton GD. The unequal variance t-test is an underused alternative to Student’s t-test and the Mann—Whitney U test. Behav Ecol. 2006;17(4):688–690. 10.1093/beheco/ark016

[37] Benjamini Y, Hochberg Y. Controlling the false discovery rate: a practical and powerful approach to multiple testing. J R Stat Soc Series B Stat Methodol. 1995; p. 289–300.

