# Supplementary material for: Integrated structural variation and point mutation signatures in cancer genomes using correlated topic models
Source: PLoS Comput Biol. 2019 Feb 22;15(2):e1006799. doi: 10.1371/journal.pcbi.1006799 (PMC6402697; doi:10.1371/journal.pcbi.1006799)
Supplement: S1 Fig — Graphical models for the a ILDA, b ICTM and c IMMCTM models, with d descriptions of their variables. See S1 Text for detailed descriptions. (PDF) [file pcbi.1006799.s001.pdf]

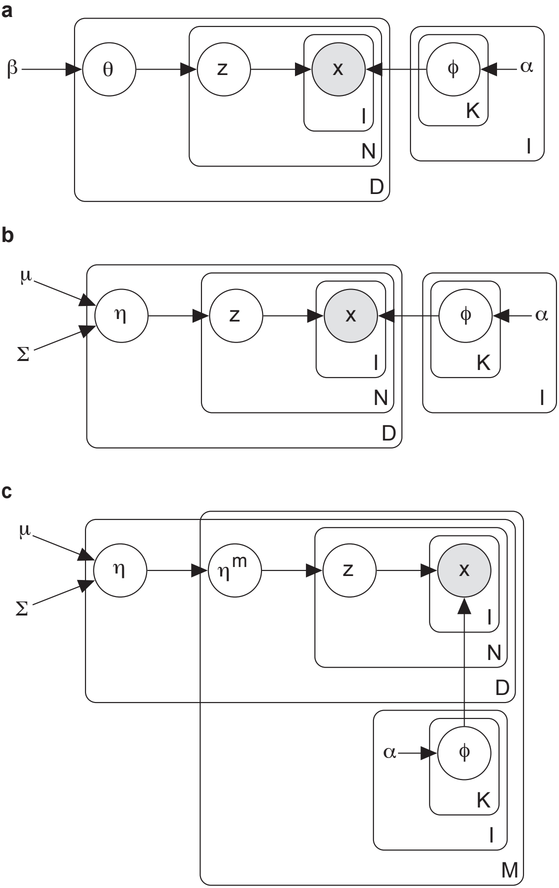

d

variable	description
β	sample-signature Dirichlet parameter
θ	sample-signature distribution
μ	Gaussian mean
Σ	Gaussian covariance
η	sample-signature unnormalized log-probabilities
η^m	modality-specific subset of η
z	mutation signature indicator
α	signature Dirichlet parameter
ϕ	signature feature distribution
x	mutation feature value
D	# samples
N	# mutations
M	# modalities
K	# signatures
I	# mutation features
