# Supplementary figures and images for: Integrated structural variation and point mutation signatures in cancer genomes using correlated topic models
Source: PLoS Comput Biol. 2019 Feb 22;15(2):e1006799. doi: 10.1371/journal.pcbi.1006799 (PMC6402697; doi:10.1371/journal.pcbi.1006799)

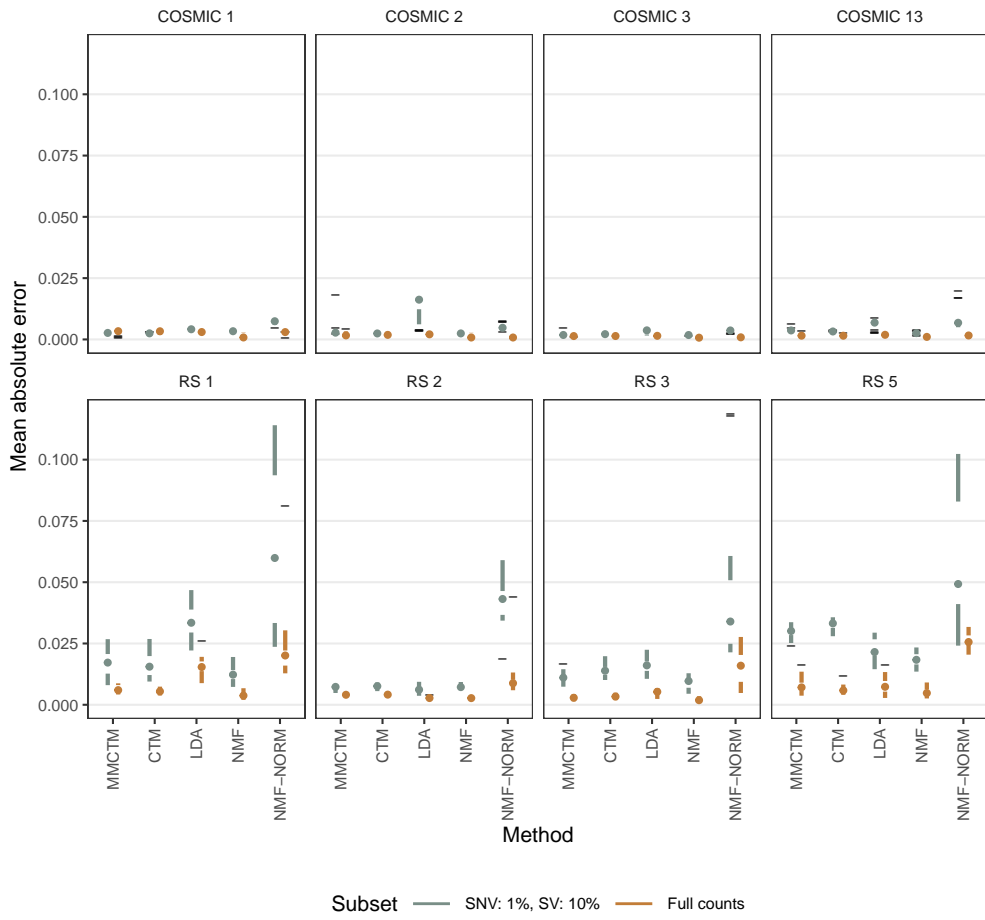

Supplement: S2 Fig — Shown are mean absolute errors per method and per signature for estimated signatures compared to the reference signatures. The experiment was repeated with full mutation counts and with 1% SNVs & 10% SVs. Data is represented as Tufte-like boxplots with the following elements: points (median), gap (first to third quartile), whisker (extends to the most extreme value no further than 1.5X the inter-quartile range from the gap edge), dash (outlier). NMF: applied to raw counts, NMF-norm: applied to normalized counts. (PDF) [file pcbi.1006799.s002.pdf]

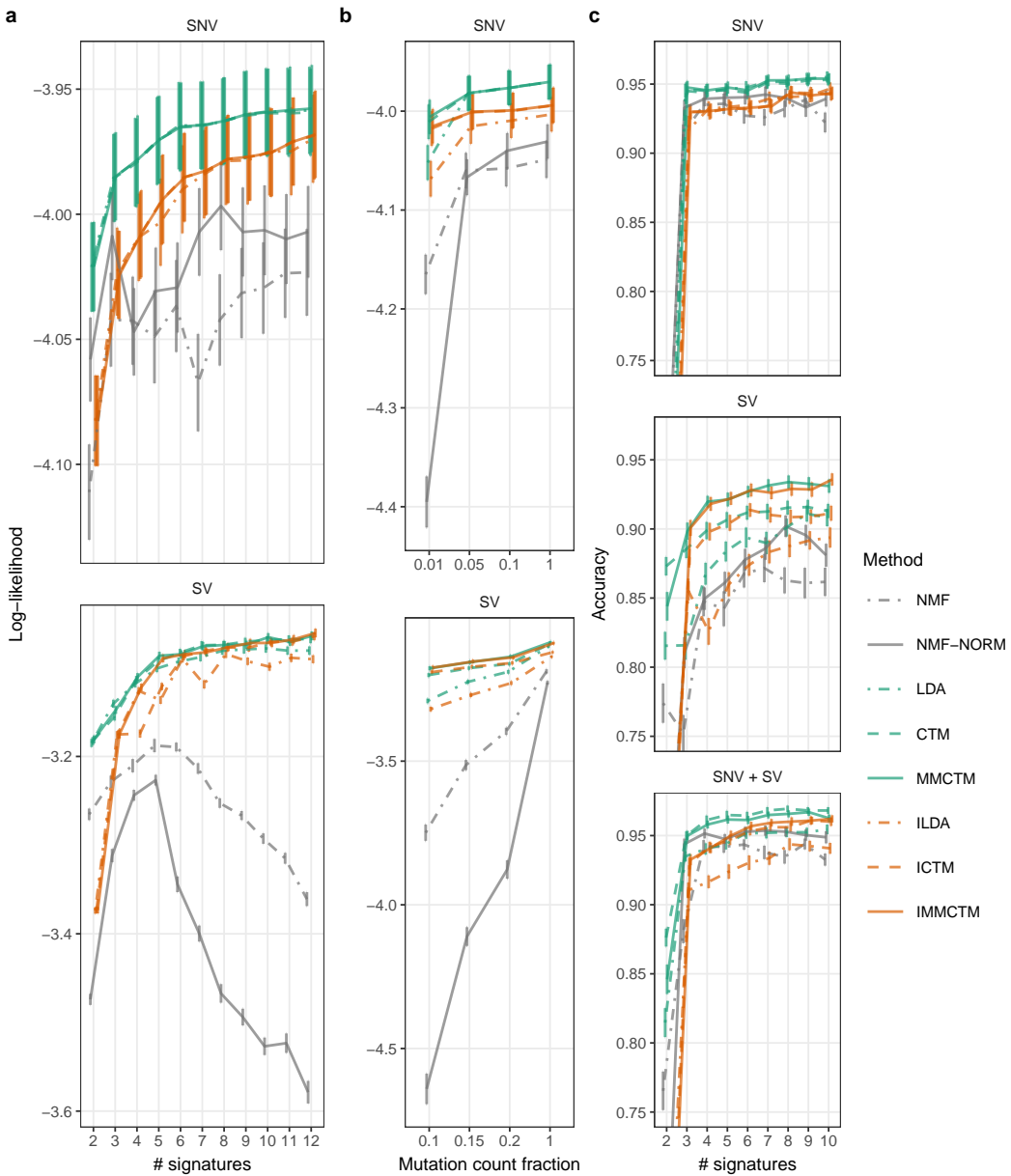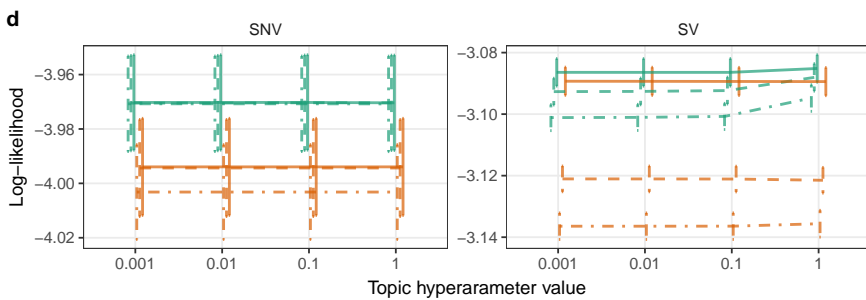

Supplement: S3 Fig — Displayed are SNV and SV signature log likelihood means ± standard error for: a 2–12 signatures, and b a range of mutation count fractions. c Logistic regression accuracy means ± standard error for predicting HRD labels using per-sample signature probabilities across a range of 1–10 signatures. d Method comparison across topic Dirichlet hyperparameter values using the breast cancer dataset. Displayed are log likelihood means ± standard error. NMF: applied to raw counts, NMF-norm: applied to normalized counts. Vertices and error bars are dodged slightly to reduce overplotting. (PDF) [file pcbi.1006799.s003.pdf]

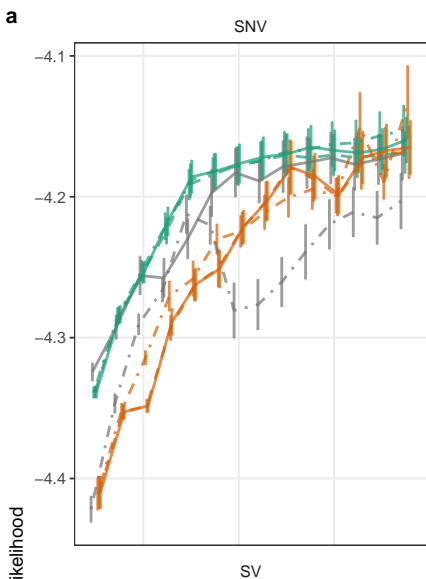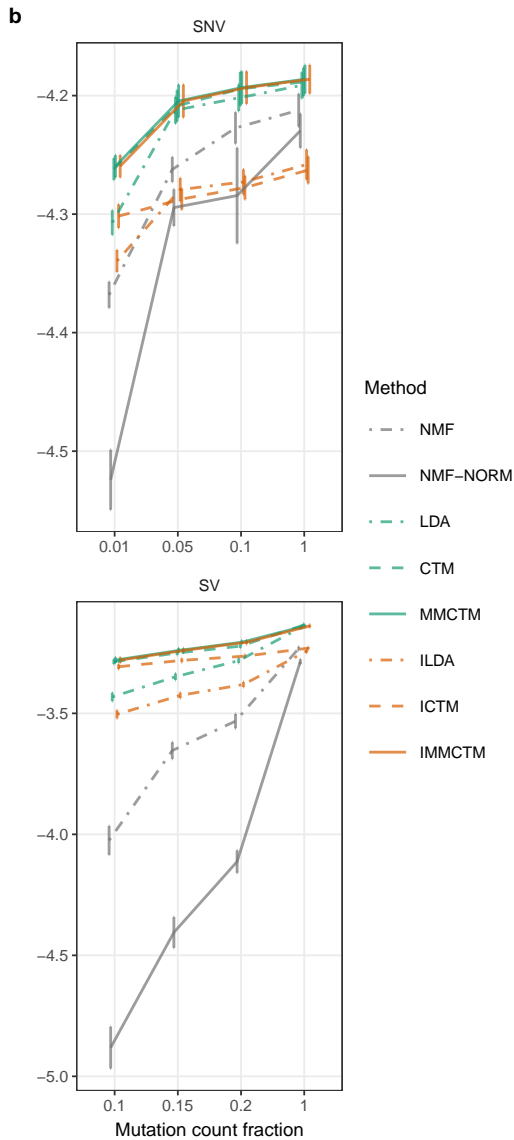

Supplement: S4 Fig — Displayed are log likelihood means ± standard error for: a 2–15 signatures, and b a range of mutation count fractions. Top panels are evaluations on SNV counts, bottom panels are evaluations on SV counts only. NMF: applied to raw counts, NMF-norm: applied to normalized counts. Downsampled SNV: only SNV counts are down-sampled. Downsampled SV: only SV counts are down-sampled. The down-sampling fractions are different for SNV and SV counts. Vertices and error bars are dodged slightly to reduce overplotting. (PDF) [file pcbi.1006799.s004.pdf]

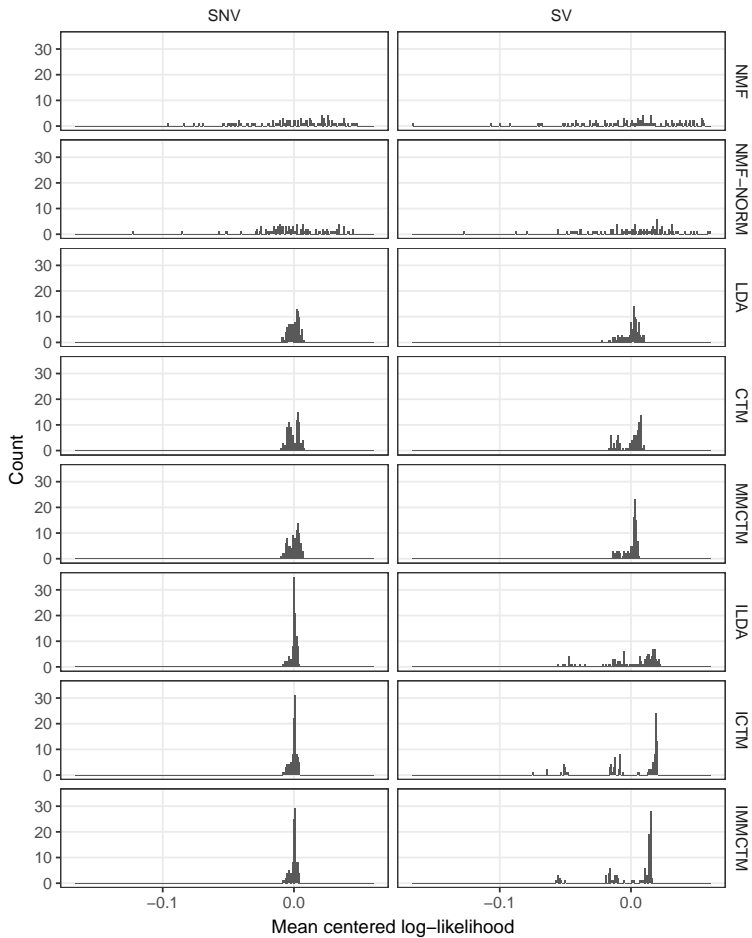

Supplement: S5 Fig — Average per-mutation predictive log-likelihoods from 100 restarts for SNV and SV signatures inferred by each method. Values have been mean-centered. (PDF) [file pcbi.1006799.s005.pdf]

**a**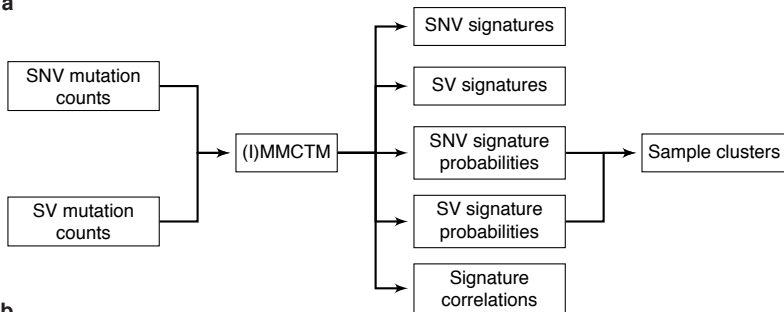**b**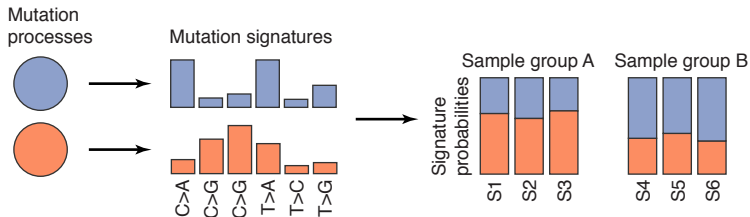

Supplement: S6 Fig — a Analysis workflow for the multimodal topic models MMCTM and IMMCTM. b Mutation process activity is detected as patterns of mutations, i.e. mutation signatures, in the genome. Samples with common levels of signature probabilities may be grouped, and potentially exhibit similar phenotypes. (PDF) [file pcbi.1006799.s006.pdf]

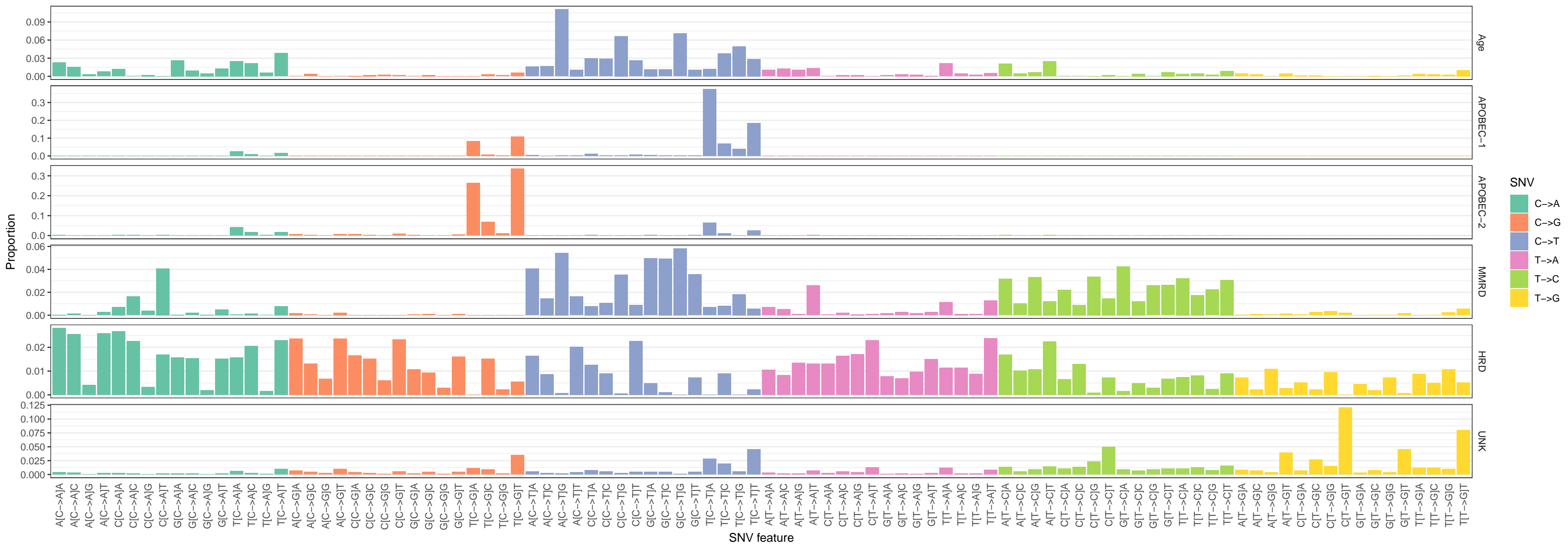

Supplement: S7 Fig — Mutation and flanking sequence shown on x-axis. (PDF) [file pcbi.1006799.s007.pdf]

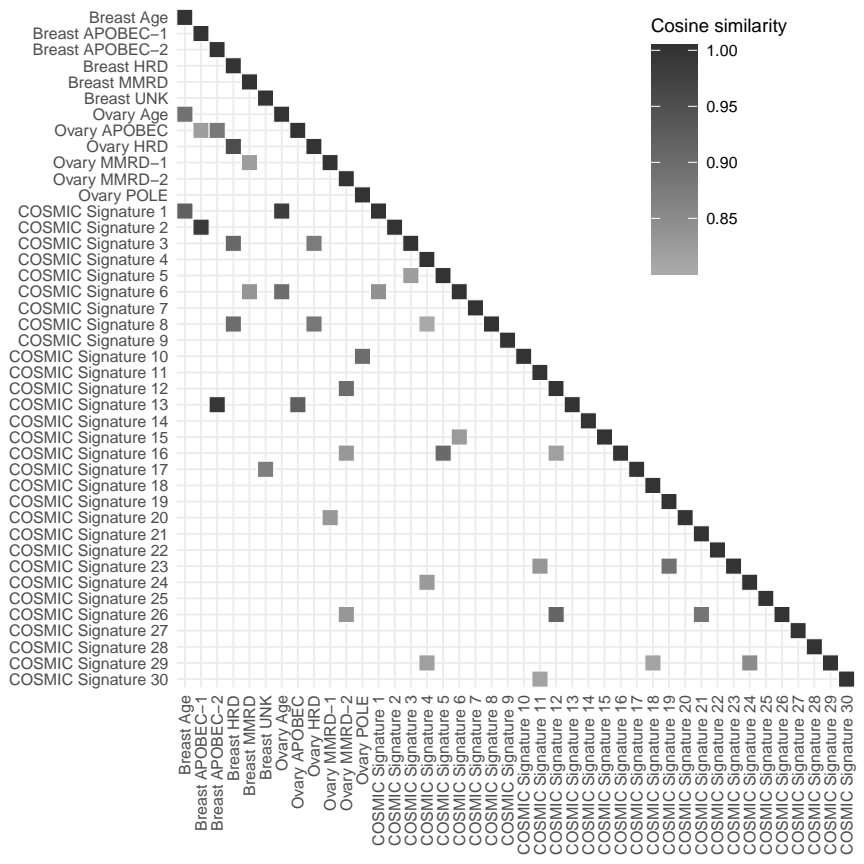

Supplement: S8 Fig — Included are SNV signatures from COSMIC, the breast, and ovarian cancer datasets. (PDF) [file pcbi.1006799.s008.pdf]

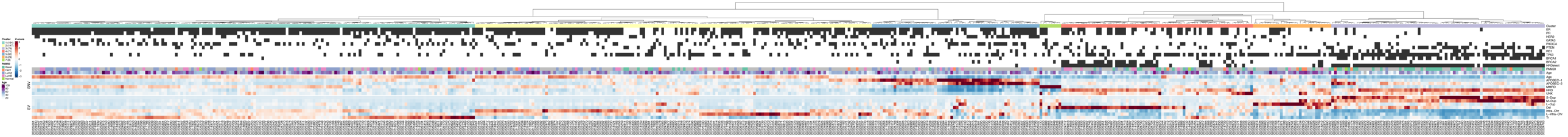

Supplement: S9 Fig — Each heatmap column represents a single sample, and is composed of the probabilities of SNV and SV signatures output from the MMCTM model. The values for each signature (row) have been standardized, producing z-scores. Heatmap display has been truncated to ±3. Samples have been hierarchically clustered according to their transformed signature probabilities and cluster labels are indicated with colours underneath the dendrogram. The number of samples in each cluster is indicated in parentheses in the cluster legend. ER, PR, and HER2 positive status are indicated with black bars. Similarly, BRCA1/2 mutation status and HRDetect prediction are indicated. (PDF) [file pcbi.1006799.s009.pdf]

**a**

Signature

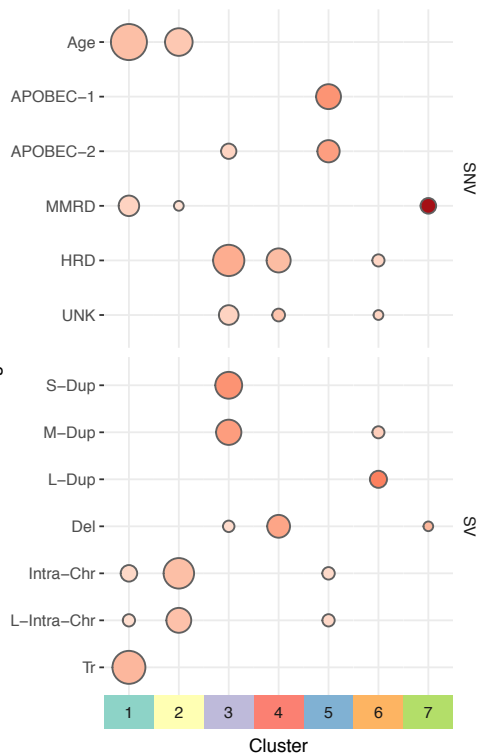**b**

Signature

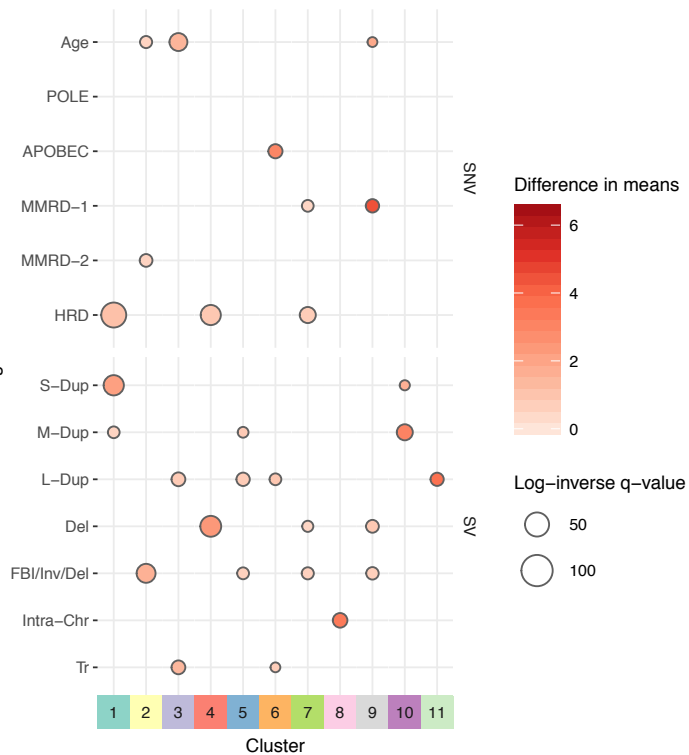

Supplement: S10 Fig — Tests compared signature probability means for clusters in the a breast, and b ovarian cancer datasets. Adjusted p-values >0.05 are not shown. Cluster labels are colored according to those in the associated signature probability heatmap. (PDF) [file pcbi.1006799.s010.pdf]

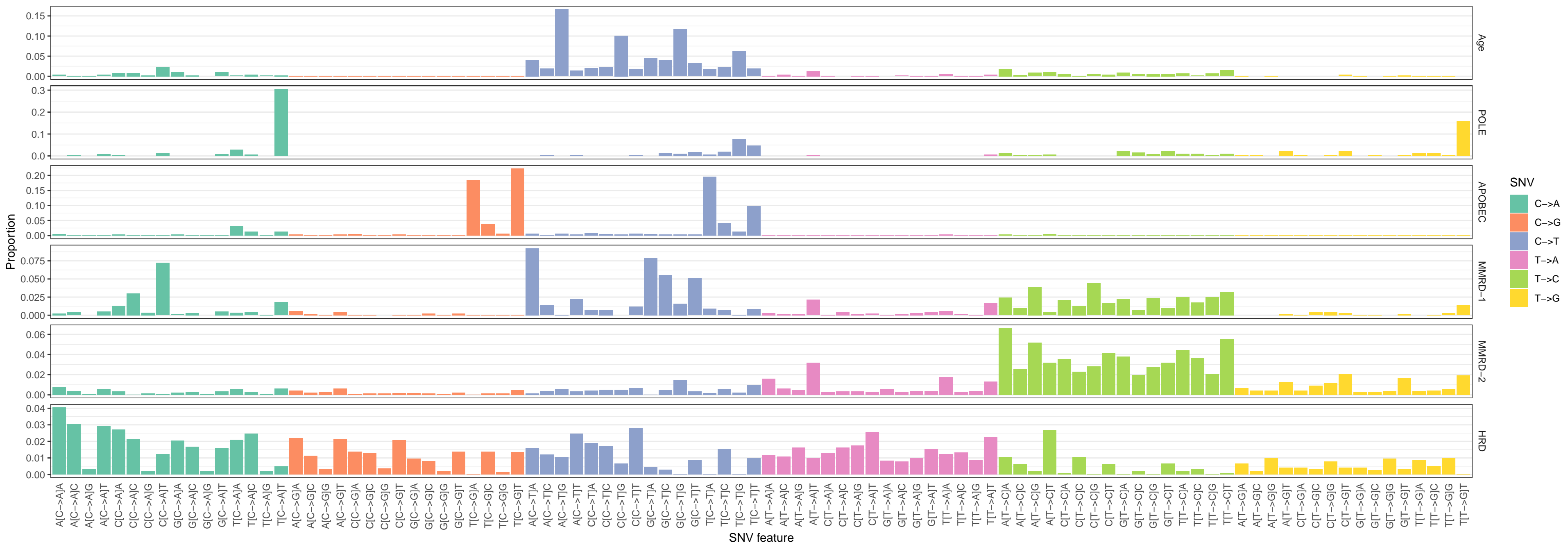

Supplement: S11 Fig — Mutation and flanking sequence shown on x-axis. (PDF) [file pcbi.1006799.s011.pdf]

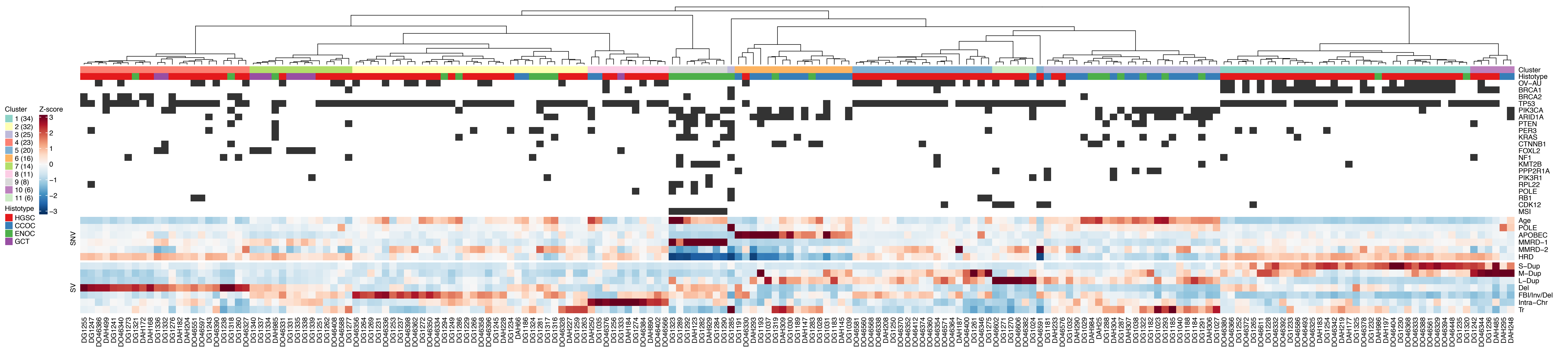

Supplement: S12 Fig — Each heatmap column represents a single sample, and is composed of the probabilities of SNV and SV signatures output from the MMCTM model. The values for each signature (row) have been standardized, producing z-scores. Heatmap display has been truncated to ±3. Samples have been hierarchically clustered according to their transformed signature probabilities and cluster labels are indicated with colours underneath the dendrogram. The number of samples in each cluster is indicated in parentheses in the cluster legend. Samples from the ICGC OV-AU project are indicated with black bars, as is gene mutation and MSI status. (PDF) [file pcbi.1006799.s012.pdf]

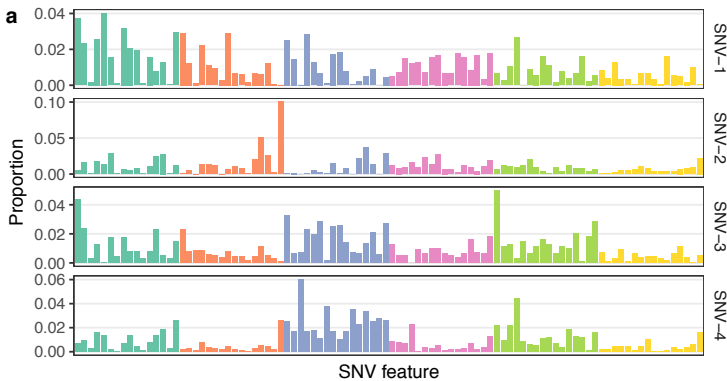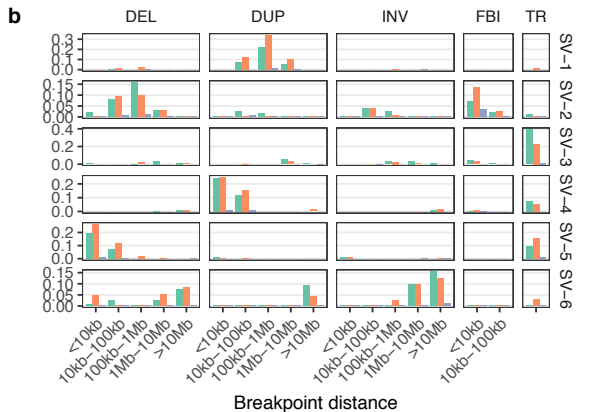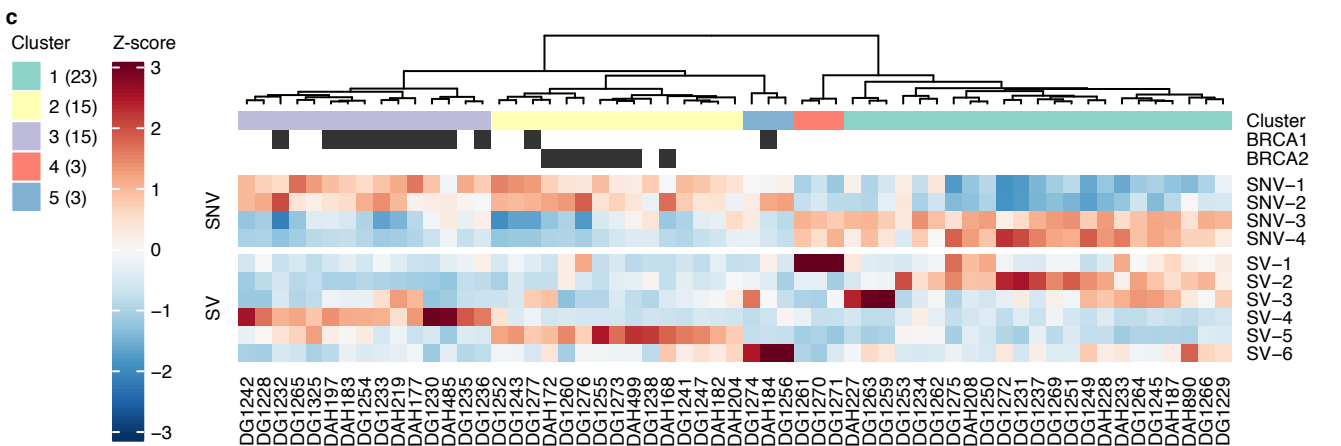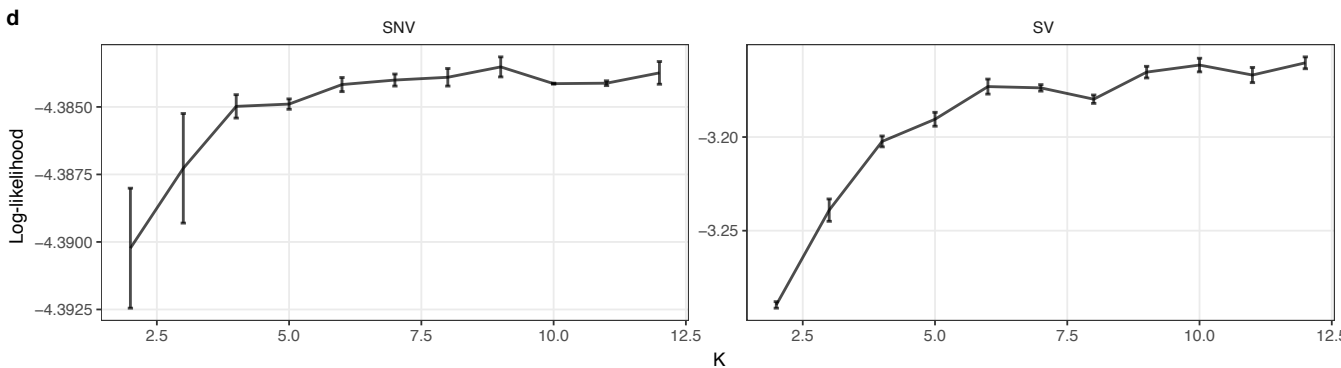

Supplement: S13 Fig — a SNV mutation signatures. SNVs are organized according to the SNV type (color). Within each type, SNVs are further organized into the pattern of flanking nucleotides (A—A, A—C, …,T—G, T—T). b SV mutation signatures. SVs are grouped by type (DEL: deletion, DUP: tandem duplication, INV: inversion, FBI: foldback inversion, TR: translocation). c Heatmap of relative signature probabilities in HGSC cancer samples. Each heatmap column represents a single sample, and is composed of the probabilities of SNV and SV signatures output from the MMCTM model. The values for each signature (row) have been standardized, producing z-scores. Heatmap display has been truncated to ±3. Samples have been hierarchically clustered according to their transformed signature probabilities and cluster labels are indicated with colors underneath the dendrogram. The number of samples in each cluster is indicated in parentheses in the cluster legend. Samples with mutated BRCA1/2 or methylated BRCA1 genes indicated with black boxes under the cluster assignments above the heatmap. d Signature log likelihood means ± standard error for 2–12 signatures. Signatures estimated from one half of counts, log-likelihood evaluated on the other half. Used to choose number of signatures. (PDF) [file pcbi.1006799.s013.pdf]

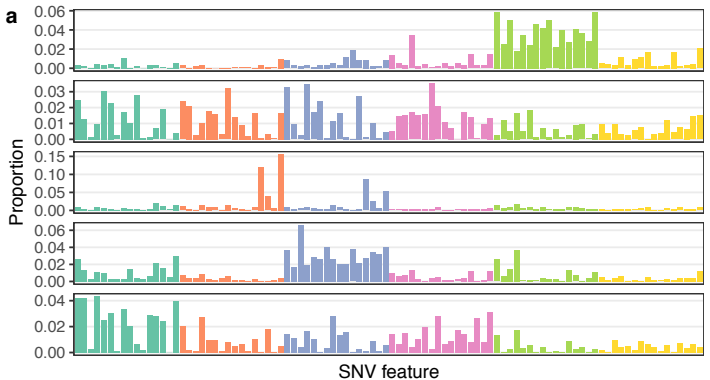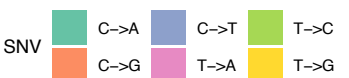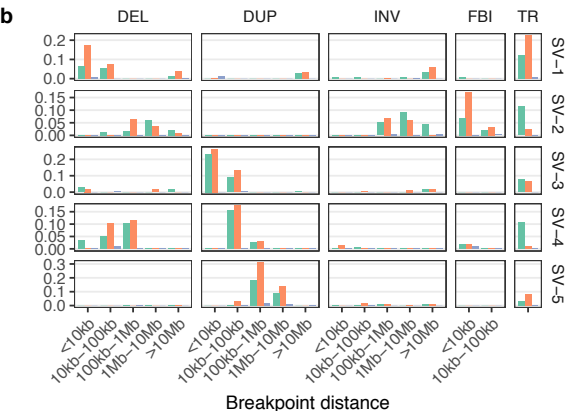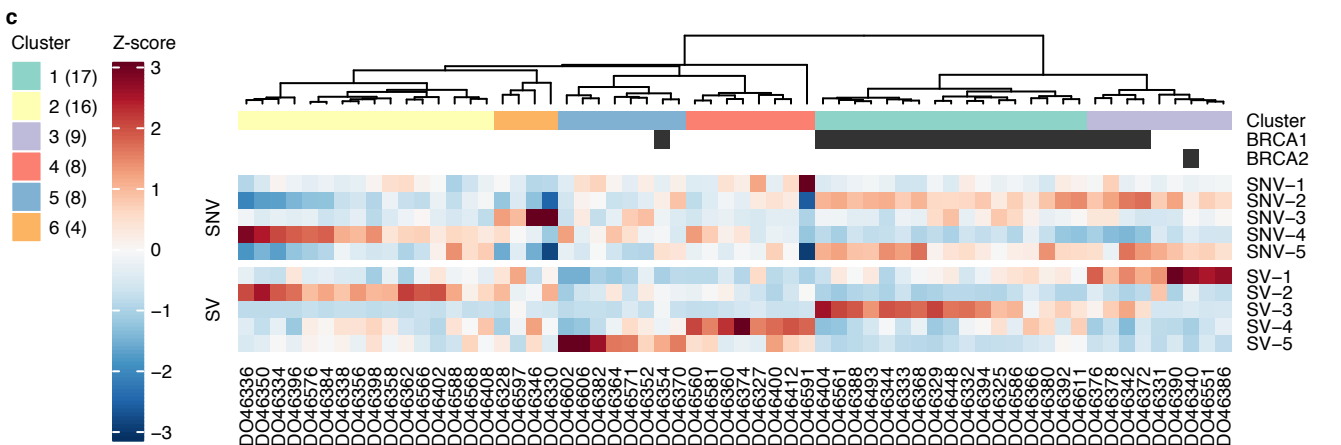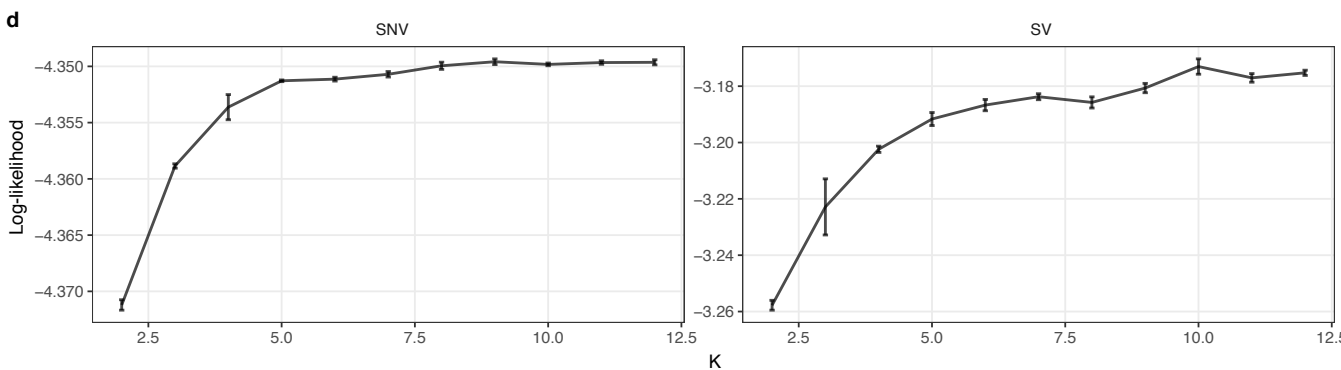

Supplement: S14 Fig — a SNV mutation signatures. SNVs are organized according to the SNV type (color). Within each type, SNVs are further organized into the pattern of flanking nucleotides (A—A, A—C, …,T—G, T—T). b SV mutation signatures. SVs are grouped by type (DEL: deletion, DUP: tandem duplication, INV: inversion, FBI: foldback inversion, TR: translocation). c Heatmap of relative signature probabilities in HGSC cancer samples. Each heatmap column represents a single sample, and is composed of the probabilities of SNV and SV signatures output from the MMCTM model. The values for each signature (row) have been standardized, producing z-scores. Heatmap display has been truncated to ±3. Samples have been hierarchically clustered according to their transformed signature probabilities and cluster labels are indicated with colors underneath the dendrogram. The number of samples in each cluster is indicated in parentheses in the cluster legend. Samples with mutated or methylated BRCA1/2 genes indicated with black boxes under the cluster assignments above the heatmap. d Signature log likelihood means ± standard error for 2–12 signatures. Signatures estimated from one half of counts, log-likelihood evaluated on the other half. Used to choose number of signatures. (PDF) [file pcbi.1006799.s014.pdf]

**a**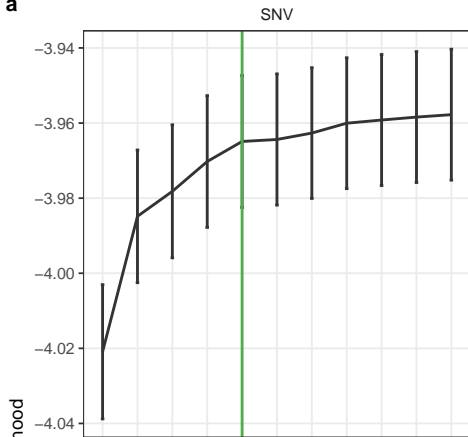

Log-likelihood

SV

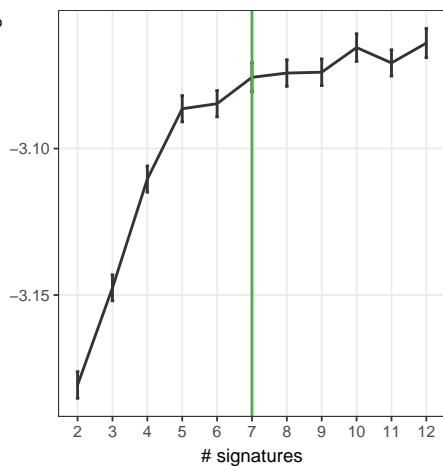**b**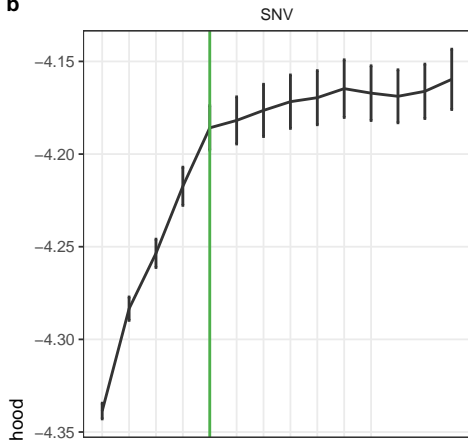

Log-likelihood

SV

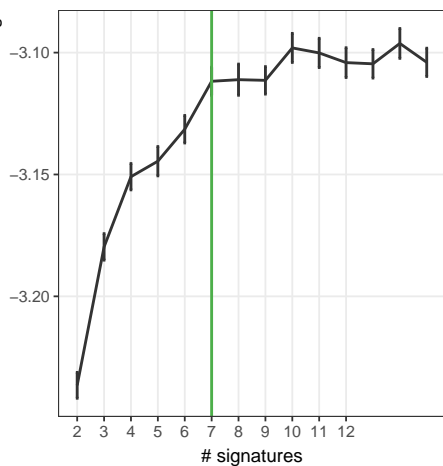

Supplement: S15 Fig — Shown for: a breast, and b ovarian cancer datasets. Signature number choice indicated as an green vertical line. (PDF) [file pcbi.1006799.s015.pdf]
