# Supplementary material for: Integrated structural variation and point mutation signatures in cancer genomes using correlated topic models
Source: PLoS Comput Biol. 2019 Feb 22;15(2):e1006799. doi: 10.1371/journal.pcbi.1006799 (PMC6402697; doi:10.1371/journal.pcbi.1006799)
Supplement: S1 Table — (PDF) [file pcbi.1006799.s016.pdf]

Description of methods

Method	Description
NMF	Factorizes a matrix with non-negative elements into two non-negative matrices. These matrices represent the unnormalized signatures and sample-signature weights.
LDA	Probabilistic topic model that represents signatures as discrete distributions, and sample-signature weights as discrete distributions drawn from a Dirichlet distribution.
CTM	An extension of LDA that treats sample-signature weights as transformed Normally-distributed variables. This model captures signature weight correlations across samples.
MMCTM	An extension of the CTM that allows multiple distinct "vocabularies". This allows the model to perform joint inference of, *e.g.*, SNV and SV signatures.
ILDA	A variation of LDA that treats mutation types and contextual information as independent features.
ICTM	An extension of the ILDA model along the lines of the CTM.
IMMCTM	An extension of the ICTM along the lines of the MMCTM.
