# Supplementary material for: Integrated structural variation and point mutation signatures in cancer genomes using correlated topic models
Source: PLoS Comput Biol. 2019 Feb 22;15(2):e1006799. doi: 10.1371/journal.pcbi.1006799 (PMC6402697; doi:10.1371/journal.pcbi.1006799)
Supplement: S2 Table — (PDF) [file pcbi.1006799.s017.pdf]

Dataset breakdown

Cancer type	# samples
Breast	560
Ovarian endometrioid	29
Ovarian clear cell	35
Ovarian granulosa cell	10
Ovarian high grade serous	121
Total Ovarian	195
Total	755
