# Supplementary material for: Integrated structural variation and point mutation signatures in cancer genomes using correlated topic models
Source: PLoS Comput Biol. 2019 Feb 22;15(2):e1006799. doi: 10.1371/journal.pcbi.1006799 (PMC6402697; doi:10.1371/journal.pcbi.1006799)
Supplement: S1 Text — (PDF) [file pcbi.1006799.s018.pdf]

Description of topic models

LDA

The generative process of latent Dirichlet allocation (LDA) is defined as:

1. draw $\phi_k \sim Dir(\alpha)$, for each signature k, where $Dir(\alpha)$ is a symmetric Dirichlet distribution with parameter α
2. draw $\theta_d \sim Dir(\beta)$, for each sample d
3. draw signature assignment, $z_{dn} \sim Cat(\theta_d)$, for each mutation n, in each sample d, where $Cat(\theta)$ is a categorical distribution with probabilities θ
4. draw $x_{dn} \sim Cat(\phi_{z_{dn}})$, for each mutation n, in each sample d

Given parameters α and β, the joint distribution of mutations X, mutation-signature assignments Z, signature mixtures θ, and signatures ϕ is given by:

$$\begin{aligned} p(X, Z, \theta, \phi \mid \alpha, \beta) = & \prod_{k=1}^{K} p(\phi_k \mid \alpha) \\ & \times \prod_{d=1}^{D} p(\theta_d \mid \beta) \prod_{n=1}^{N_d} p(z_{dn} \mid \theta_d) p(x_{dn} \mid z_{dn}, \phi) \end{aligned} \tag{1}$$

where K is the number of signatures, D is the number of samples, N_d is the number of mutations in a sample, d.

CTM

The correlated topic model (CTM) modifies LDA by replacing the θ variable with a K-dimensional Normally-distributed variable η. The Gaussian covariance matrix captures signature correlations across samples. The generative process is:

1. draw $\phi_k \sim Dir(\alpha)$, for each signature k
2. draw $\eta_d \sim N(\mu, \Sigma)$, for each sample d, where $N(\mu, \Sigma)$ is the multivariate Gaussian distribution with mean μ and covariance Σ
3. draw signature assignment, $z_{dn} \sim Cat(f(\eta_{d,1..K}))$, for each mutation n, in each sample d, where $f(\eta_{dk}) = \frac{exp(\eta_{dk})}{\sum_{k'}^{K} exp(\eta_{dk'})}$ transforms $\eta_{d,1..K}$ to a valid probability distribution
4. draw $x_{dn} \sim Cat(\phi_{z_{dn}})$, for each mutation n, in each sample d

The joint probability then becomes:

$$\begin{aligned} p(X, Z, \eta, \phi \mid \alpha, \mu, \Sigma) = & \prod_{k=1}^{K} p(\phi_k \mid \alpha) \\ & \times \prod_{d=1}^{D} p(\eta \mid \mu, \Sigma) \prod_{n=1}^{N_d} p(z_{dn} \mid \eta_d) p(x_{dn} \mid z_{dn}, \phi) \end{aligned} \tag{2}$$

where K is the number of signatures, D is the number of samples, N_d is the number of mutations in a sample d.

MMCTM

The multi-modal correlated topic model (MMCTM) extends the CTM further, by allowing signature inference for multiple data/mutation types (*i.e.* modalities) simultaneously. The generative process is as follows:

1. draw $\phi_k^m \sim Dir(\alpha^m)$, for each signature k, in each modality m
2. draw $\eta_d \sim N(\mu, \Sigma)$, for each sample d, where $N(\mu, \Sigma)$ is the multivariate Gaussian distribution with mean μ and covariance Σ
3. draw signature assignment, $z_{dn}^m \sim Cat(f(\eta_{d,1..K^m}^m))$, for each mutation n, in each sample d, in each modality m, where η_d^m is a modality-specific subset of η_d, *i.e.* $\eta_d = \eta_d^1, \ldots, \eta_d^M$
4. draw $x_{dn}^m \sim Cat(\phi_{z_{dn}}^m)$, for each mutation n, in each sample d, in each modality m

The joint probability is written as:

$$\begin{aligned} p(X, Z, \eta, \phi \mid \alpha, \mu, \Sigma) = & \prod_{m=1}^{M} \prod_{k=1}^{K^m} p(\phi_k^m \mid \alpha^m) \\ & \times \prod_{d=1}^{D} p(\eta_d \mid \mu, \Sigma) \prod_{m=1}^{M} \prod_{n=1}^{N_d^m} p(z_{dn}^m \mid \eta_d^m) p(x_{dn}^m \mid z_{dn}^m, \phi^m) \end{aligned} \tag{3}$$

where M is the number of modalities, K^m is the number of signatures in a modality m, D is the number of samples, N_d^m is the number of mutations in a sample modality. When using only a single modality, the MMCTM reduces to the CTM. Therefore, CTM parameters were inferred using the MMCTM implementation in this study, but with counts from a single mutation type.

IMMCTM

The independent-feature multi-modal correlated topic model (IMMCTM) is based on a previously described independent mutation feature model [1], as well as the MMCTM [2,3]. This model represents signatures as a collection of independent mutation features. For example, like typical SNV signatures [4] both the substitution and flanking nucleotides can be included in a signature definition; however, this model can treat the substitution and flanking nucleotides independently rather than concatenating them into a combined mutation type. This means that for signatures taking into account trinucleotide content around substitutions, there can be three features (one substitution and two flanking nucleotides) with $6 + 4 + 4 = 14$ possible feature values as opposed to the typical $6 * 4 * 4 = 96$ possible feature values commonly used for mutation signatures. The generative process for the IMMCTM is as follows:

1. draw $\phi_{ki}^m \sim Dir(\alpha_i^m)$, for each feature i, in each signature k, in each modality m
2. draw $\eta_d \sim N(\mu, \Sigma)$, for each sample d
3. draw mutation signature assignment $z_{dn}^m \sim Cat(f(\eta_{d,1..K^m}^m))$, for each mutation n, in each sample d, in each modality m
4. draw $x_{dni}^m \sim Cat(\phi_{z_{dn}^m i}^m)$, for each mutation feature i, in each mutation n, in each modality m, in each sample d

Then the model joint probability is

$$
\begin{aligned}
p(X, Z, \eta, \phi \mid \alpha, \mu, \Sigma) = & \prod_{m=1}^{M} \prod_{k=1}^{K^m} \prod_{i=1}^{I^m} p(\phi_{ki}^m \mid \alpha_i^m) \\
& \times \prod_{d=1}^{D} p(\eta^d \mid \mu, \Sigma) \prod_{m=1}^{M} \prod_{n=1}^{N_d^m} p(z_{dn}^m \mid \eta_d^m) \prod_{i=1}^{I^m} p(x_{dni}^m \mid \phi_{z_{dn}^m i})
\end{aligned}
\tag{4}
$$

where M is the number of modalities, K^m is the number of signatures in a modality m, D is the number of samples, N_d^m is the number of mutations in a sample modality, and I^m is the number of mutation features in a modality.

When using only a single modality, the IMMCTM reduces to the ICTM. Therefore, ICTM parameters were inferred using the IMMCTM implementation in this study, but with counts from a single mutation type. A similar procedure can be used to modify LDA to form ILDA, which is similar to the model described by Shiraishi et al. [1], and to modify the CTM to form the ICTM.

Inference

IMMCTM updates are similar to those for the MMCTM [3], with modifications to allow for the independent feature construction of the mutation.

The factorized mean-field variational Bayesian approximation for the IMMCTM is

$$
\begin{aligned}
q(\eta, \phi, Z \mid \lambda, \nu, \theta, \gamma) = & \prod_{m=1}^{M} \prod_{k=1}^{K^m} \prod_{i=1}^{I^m} q(\phi_{ki}^m \mid \gamma_{ki}^m) \\
& \times \prod_{d=1}^{D} \prod_{m=1}^{M} \prod_{k=1}^{K^m} q(\eta_{dk}^m \mid \lambda_{dk}^m, \nu_{dk}^m) \\
& \times \prod_{d=1}^{D} \prod_{m=1}^{M} \prod_{n=1}^{N_d^m} q(z_{dn}^m \mid \theta_{dn}^m)
\end{aligned}
\tag{5}
$$

where

- $\phi_{ki}^m \sim Dir(\gamma_{ki}^m)$
- $\eta_{dk}^m \sim N(\lambda_{dk}^m, \nu_{dk}^m)$

- $z^m_{dn} \sim Cat(\theta^m_{dn})$

The update for γ^m_{kij} is

$$\gamma^m_{kij} = \alpha^m_i + \sum_{d=1}^{D} \sum_{n=1}^{N^m_d} \theta^m_{dnk} \, \mathbb{I}(x^m_{dni} = j) \tag{6}$$

And the update for θ^m_{dnk} is

$$\theta^m_{dnk} \propto exp\left(\lambda^m_{dk} + \sum_{i=1}^{I^m} \mathbb{E}_q[\log \phi^m_{kix^m_{dni}}]\right) \tag{7}$$

where the calculation of $\mathbb{E}_q[\log \phi^m_{kix^m_{dni}}]$ is as described in Blei *et al.* [5]

References

1. Shiraishi Y, Tremmel G, Miyano S, Stephens M. A simple model-based approach to inferring and visualizing cancer mutation signatures. PLoS genetics. 2015;11(12):e1005657.

2. Blei D, Lafferty J. Correlated topic models. Advances in neural information processing systems. 2006;18:147.

3. Salomatin K, Yang Y, Lad A. Multi-field Correlated Topic Modeling. In: SDM. SIAM; 2009. p. 628–637.

4. Alexandrov LB, Nik-Zainal S, Wedge DC, Aparicio SA, Behjati S, Biankin AV, et al. Signatures of mutational processes in human cancer. Nature. 2013;500(7463):415–421.

5. Blei DM, Ng AY, Jordan MI. Latent dirichlet allocation. Journal of machine Learning research. 2003;3(Jan):993–1022.
